# Coupling Water‐Proof Li Anodes with LiOH‐Based Cathodes Enables Highly Rechargeable Lithium–Air Batteries Operating in Ambient Air

**DOI:** 10.1002/advs.202103760

**Published:** 2021-12-11

**Authors:** Jiang Lei, Zongyan Gao, Linbin Tang, Li Zhong, Junjian Li, Yue Zhang, Tao Liu

**Affiliations:** ^1^ Shanghai Key Laboratory of Chemical Assessment and Sustainability School of Chemical Science and Engineering Tongji University No. 1239, Siping Road Shanghai 200092 P. R. China; ^2^ SEU‐FEI Nano‐Pico Center Key Laboratory of MEMS of Ministry of Education Southeast University Nanjing 210096 P. R. China

**Keywords:** lithium–air batteries, lithium anode protection, lithium hydroxide, H_2_O/CO_2_ resistance

## Abstract

Realizing an energy‐dense, highly rechargeable nonaqueous lithium–oxygen battery in ambient air remains a big challenge because the active materials of the typical high‐capacity cathode (Li_2_O_2_) and anode (Li metal) are unstable in air. Herein, a novel lithium–oxygen full cell coupling a lithium anode protected by a composite layer of polyethylene oxide (PEO)/lithium aluminum titanium phosphate (LATP)/wax to a LiOH‐based cathode is constructed. The protected lithium is stable in air and water, and permits reversible, dendrite‐free lithium stripping/plating in a wet nonaqueous electrolyte under ambient air. The LiOH‐based full cell reaction is immune to moisture (up to 99% humidity) in air and exhibits a much better resistance to CO_2_ contamination than Li_2_O_2_, resulting in a more consistent electrochemistry in the long term. The current approach of coupling a protected lithium anode with a LiOH‐based cathode holds promise for developing a long‐life, high‐energy lithium–air battery capable of operating in the ambient atmosphere.

## Graphical abstract

A highly rechargeable lithium‐air battery operating directly in ambient environment is successfully demonstrated by coupling a LiOH‐based cathode and an air‐stable lithium anode with a composite protective layer. Key advantages of the system include: 1) outstanding electrochemical stability; 2) Li dendrite‐free; 3) superior resistance to humidity (99%) and CO2; 4) being applicable to highly wet electrolytes (up to 50vol%).

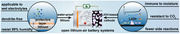

## 1 Introduction

**Figure 1:**
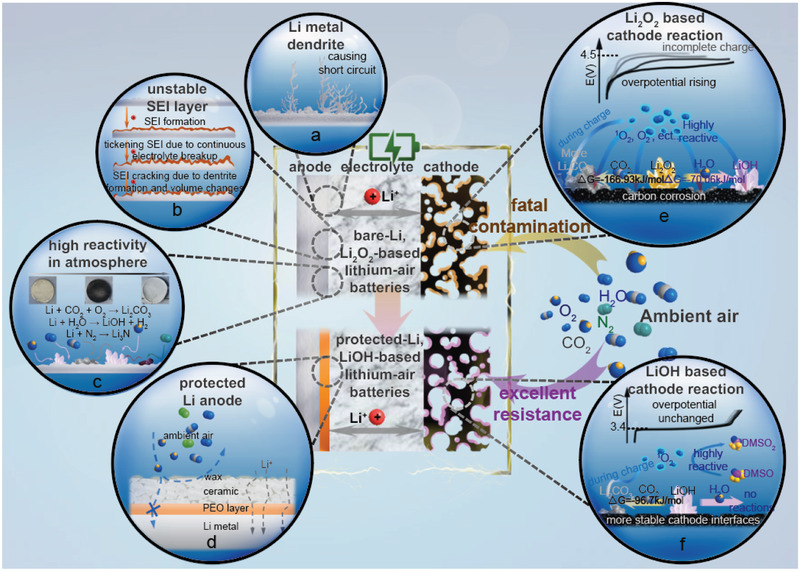
A scheme illustrating the challenges associated with a nonaqueous lithium–air battery using a bare Li anode and a Li_2_O_2_‐based cathode, and strategies implemented in this work to address these issues. a, b) Conventional issues of dendrite formation and unstable SEI for Li metal anodes. c) Many constitutes of air could corrode Li metal in an open lithium–air battery. d) Schematics of the Li anode protection strategy developed in the current work that effectively suppresses unwanted parasitic reactions in an open lithium–air environment. A comparison of the potential characteristic processes involved in Li_2_O_2_‐based e) and LiOH‐based f) lithium–air batteries.

Fully realizing the high energy density of nonaqueous lithium–air batteries relies on the use of a Li metal anode paired with a lithium oxide cathode, e.g., Li_2_O_2_ (2.96 V, 3.5 kWh kg^−1^) or Li_2_O (2.91 V, 5.2 kWh kg^−1^) in a cell operating in ambient air.^[^ ^1^, ^2^, ^3^, ^4^ ^]^ But none of these electrode materials are air stable. Li_2_O_2_ and Li_2_O would spontaneously react with H_2_O and CO_2_ to form LiOH and eventually Li_2_CO_3_,^[^ ^3^, ^5^, ^6^, ^7^, ^8^, ^9^, ^10^ ^]^ the latter inducing high overpotentials on recharging^[^ ^8^, ^11^, ^12^ ^]^ and causing further parasitic reactions to carbon cathodes^[^ ^13^, ^14^, ^15^ ^]^ and electrolytes^[^ ^11^, ^16^, ^17^ ^]^ (**Figure** **1**e). Li metal suffers from reactivity to an even wider range of air components (N_2_, O_2_, CO_2_, H_2_O),^[^ ^3^ ^]^ especially moisture,^[^ ^18^ ^]^ alongside the conventional problems pertaining to an unstable solid‐electrolyte interphase (SEI) and Li dendrite formation (Figure 1a–c).^[^ ^19^, ^20^, ^21^, ^22^ ^]^ These issues pose major challenges to the realization of a high‐energy lithium–air battery in practice.

To achieve stable electrochemical Li stripping/plating, many previous studies have employed solid‐state Li^+^ conductive ceramics in a dual compartmental design for lithium–air batteries.^[^ ^23^, ^24^, ^25^, ^26^, ^27^ ^]^ These nonporous discs of solid Li^+^ conductors can be water‐ and air‐proof,^[^ ^28^, ^29^, ^30^, ^31^, ^32^ ^]^ but they tend to degrade when in direct contact with Li metal^[^ ^33^, ^34^ ^]^ and are very susceptible to crack formation, where Li dendrites and air could readily penetrate through.^[^ ^34^, ^35^ ^]^ Some studies involve uses of organic‐inorganic composite films,^[^ ^36^, ^37^, ^38^ ^]^ which tend to be more flexible and uniform on Li and have been shown to greatly improve the electrochemical stability of Li anodes. Nevertheless, in some of these cases Li dendrites still form, and are prone to penetrating through the softer organic component of the protective film.^[^ ^39^ ^]^ Other relevant lithium–air battery studies using protected lithium anodes have either examined the cell performance under a pure O_2_ atmosphere, and with little/no water in lithium‐compatible electrolytes.^[^ ^40^, ^41^, ^42^, ^43^, ^44^, ^45^, ^46^, ^47^ ^]^ It remains challenging to achieve reversible, dendrite‐free Li stripping/plating using a wet nonaqueous electrolyte in an open (humidified) lithium–air battery.

At the cathode, LiOH being an alternative active material, is theoretically immune to moisture and more resistant to CO_2_ contamination compared to Li_2_O_2_ and Li_2_O (Figure 1f).^[^ ^3^ ^]^ Recently, LiOH formation and decomposition (3.32 V, 3.7 kWh kg^−1^) have been demonstrated as reversible four‐electron oxygen reduction (ORR) and evolution reactions (OER), i.e.

(1) $$4Li^{+}+4e^{-}+O_{2}+2H_{2}O\to 4\mathrm{LiOH}$$

in a variety of catalytic systems in the presence of water; these include systems involving the uses of LiI mediator,^[^ ^48^, ^49^ ^]^ Cu^I^‐complex enzyme,^[^ ^50^ ^]^ sodium trifluoromethanesulfonate^[^ ^51^ ^]^ as additives in nonaqueous electrolytes or the use of solid catalysts (Co_3_O_4_, Mn‐MOF‐74, activated graphene).^[^ ^52^, ^53^, ^54^ ^]^ However, no study so far has evaluated the electrochemical stability of a LiOH‐based cell reaction in ambient air, and investigated whether or how H_2_O and CO_2_ in air would alter the nature of the reaction product.

Herein, we use Ru‐catalyzed LiOH formation/decomposition reaction as a model system to assess the suitability of using LiOH as the cathode active material in an open lithium–air battery. This Ru‐based system is simple, avoiding potential complicating cell reactions involving O_2_, H_2_O, CO_2_ with mediator or enzyme additives and shuttling of these redox species;^[^ ^55^, ^56^, ^57^ ^]^ it also exhibits a consistent LiOH‐based electrochemistry throughout the whole capacity with low overpotentials and relatively good rechargeability.^[^ ^58^ ^]^ As such, it allows a true assessment of air effects on the stability of this cathode electrochemistry in both short and long terms. In addition, we have designed a hierarchical multifunctional layer on the Li anode to enable stable operation of a full battery in ambient air (Figure 1d). Our work demonstrates that the protected lithium anode is chemically stable in air and in pure water; the resulting full lithium–air cell using the protected Li anodes and LiOH‐based cathodes shows an excellent electrochemical stability, operating even in an electrolyte with a substantial amount of water, successfully addressing many of the aforementioned issues that plagued lithium–air battery development.

A variety of analysis techniques, including *operando* electrochemical mass spectrometry (OEMS), chemical titration, X‐ray diffraction (XRD), solid state nuclear magnetic resonance (ssNMR), Fourier transformed infrared spectroscopy (FTIR), X‐ray photon‐electron spectroscopy (XPS), and so on (see Methods, Figures S1–S4, Supporting Information) have been employed to demonstrate and rationalize the superior electrochemical stability. We anticipate that when a fully reversible chemistry based on LiOH formation/decomposition is utilized at the cathode, the cell cycling life could be improved even further. In the ensuing sections, the effect of air on the cathode electrochemistry is first discussed, followed by the demonstration of the Li protection strategy that enables full‐cell operation.

## 2 Results and Discussion

**Figure 2:**
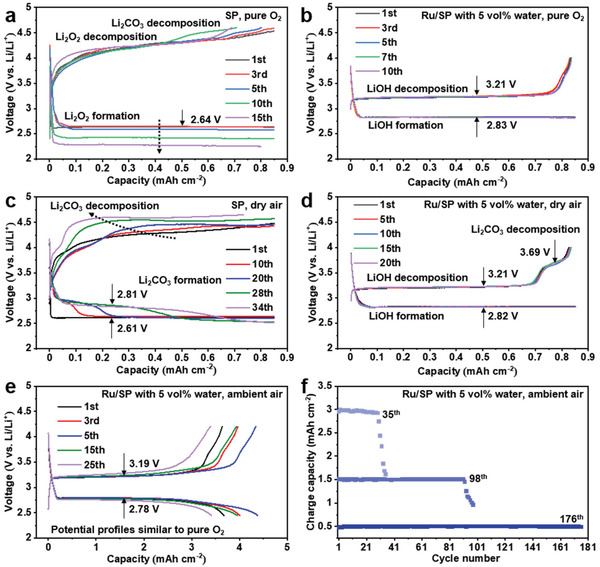
Electrochemical performance of Li‐O_2_/air cells using different cathodes and electrolytes. Voltage profiles of cells using SP cathodes and 1 m LiTFSI/DMSO in pure O_2_ (a) and in dry air (c); cell voltage profiles using Ru/SP cathodes and 1 m LiTFSI/DMSO with 5 vol% H_2_O in pure O_2_ (b) and in dry air (d). Cells in a‐d) are cycled at 0.425 mA cm^−2^. Electrochemical profiles of cells e) using Ru/SP and 1 m LiTFSI/DMSO with 5 vol% in ambient air, and long‐term cyclability of these cells f) with different depths of discharge at 0.5, 1.5 and 3 mAh cm^−2^ (or 3000 mAh g_c_^−1^, as the SP cathode size and loading are typically around 0.5 cm^2^ and 1 mg cm^−2^). The specific capacities could be a few times higher if carbon nanotubes are used to replace SP carbon in cathodes.^[^ ^62^, ^63^ ^]^

**Figure** **2** compares the effects of operating atmospheres (i.e., pure O_2_, dry air, ambient air) on the electrochemistry of two cell systems: one uses Super P (SP) carbon as the cathode and anhydrous Lithium bis(trifluoromethanesulfonyl)imide in dimethyl sulfoxide (LiTFSI/DMSO) as the electrolyte, and the other uses a ruthenium decorated SP (Ru/SP) cathode (Figure S5, Supporting Information) and a wet LiTFSI/DMSO electrolyte with 5 vol% added water. These two systems are expected to form a Li_2_O_2_‐ and LiOH‐dominant electrochemistry, respectively.^[^ ^58^, ^59^, ^60^ ^]^ Air‐stable lithium iron phosphate was used as the counter/reference electrode to help focus on investigating the half‐cell reaction at the air cathode.

In pure O_2_ (Figure 2a), the cell showed discharge and charge plateaus at ≈2.6 and ≈4.2 V, respectively, which are consistent with the processes of Li_2_O_2_ formation and decomposition reported in literature.^[^ ^59^, ^60^, ^61^ ^]^ After only a few cycles, the discharge overpotential started to increase and meanwhile a new charge plateau at ≈4.4 V emerged; the rises in overpotentials rapidly led to premature discharge and charge processes at the cutoff voltages and failure of the cell. Using a Ru/SP cathode, on the other hand, the cell revealed (Figure 2b) an overlapping electrochemical profile with high Coulombic efficiencies (>99%) over extended cycles. Flat discharge and charge voltages at ≈2.8 and ≈3.2 V were observed, which has been ascribed to LiOH formation and decomposition, respectively.^[^ ^58^ ^]^

Switching to a dry air (21.2% O_2_, 0.038% CO_2_, 78.76% N_2_) atmosphere caused a few notable changes to the electrochemistry of cells using SP cathodes (Figure 2c): a new discharge plateau at ≈2.8 V appeared and elongated with cycle number; the charging voltage continually rose and the plateau at 4.4 V rapidly became the dominant feature in the charge profile, approaching the cutoff voltage set at 4.65 V.^[^ ^8^, ^64^, ^65^ ^]^ In Ru/SP cells, however, the electrochemical profile was largely unchanged (Figure 2d), the voltage gaps and consistency between cycles remaining the same. But a small new charge plateau did develop at ≈3.65 V, its length staying almost constant with cycling. Figure 2e shows the electrochemistry of a Ru/SP cell that was fully discharged and charged in ambient air, with an average capacity of 3.7 mAh cm^−2^ in the first 25 cycles. Its electrochemical profile bears an even higher resemblance to the one in pure O_2_, with the 3.65 V plateau barely discernable. Long‐term rechargeability in ambient air with each cycle curtailed to different capacities is illustrated in Figure 2f. Areal capacities are being quoted due to its more practical relevance. Typically, a cell can cycle in ambient air at 1.5 mAh cm^−2^ (1500 mAh g_c_^−1^) per cycle for around 100 times, before the capacity starts to fade. The above data demonstrate that the Ru/SP‐catalyzed reaction at the cathode possesses a higher electrochemical stability and better rechargeability in air than the typical Li_2_O_2_‐based cell reaction.

It is worth mentioning that we have also investigated the electrochemistry of cells using Ru/SP cathodes and *anhydrous* electrolytes in a strictly sealed chamber; the cells showed an electrochemical profile (see detailed discussion in Figure S6, Supporting Information) gradually converting to that in wet electrolytes (Figure 2d). Quantitative analysis indicates that the reaction changed from close to two electrons per reduced O_2_ (i.e., consistent with Li_2_O_2_) to approximately four electrons per O_2_ (i.e., LiOH); this mixed and evolving electrochemistry using a Ru/SP cathode hinders a clear‐cut comparison of air resistance between the Li_2_O_2_‐ and LiOH‐based systems, and thus a SP cathode, instead, has been used for comparison.

**Figure 3:**
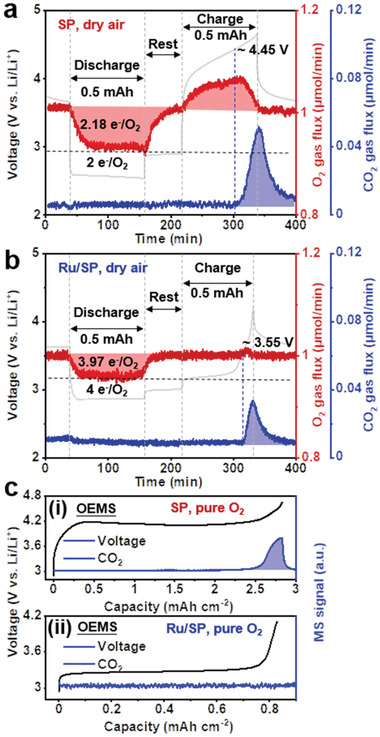
Gases consumption and evolution during cell cycling in air using operando electrochemical mass spectrometry (OEMS). Discharge and charge profiles and the corresponding O_2_ (*m/z* = 32), CO_2_ (*m/z* = 44) signals of cells using different cathodes and electrolytes under dry air: a) SP cathode with 1 m LiTFSI/DMSO and b) Ru/SP cathode with 1 m LiTFSI/DMSO with 5 vol% H_2_O. The blue dashed line denotes the onset of CO_2_ release in the two systems. Levels for a) two electrons per reduced O_2_ and b) four electrons per reduced O_2_ are marked using black dashed lines. c) Comparison of CO_2_ (*m/z* = 44) evolved during electrochemical decomposition of Li_2_O_2_‐ (SP, anhydrous 1 m LiTFSI/DMSO) and LiOH‐based (Ru/SP, 1 m LiTFSI/DMSO with 5 vol% H_2_O) battery systems.

**Figure 4:**
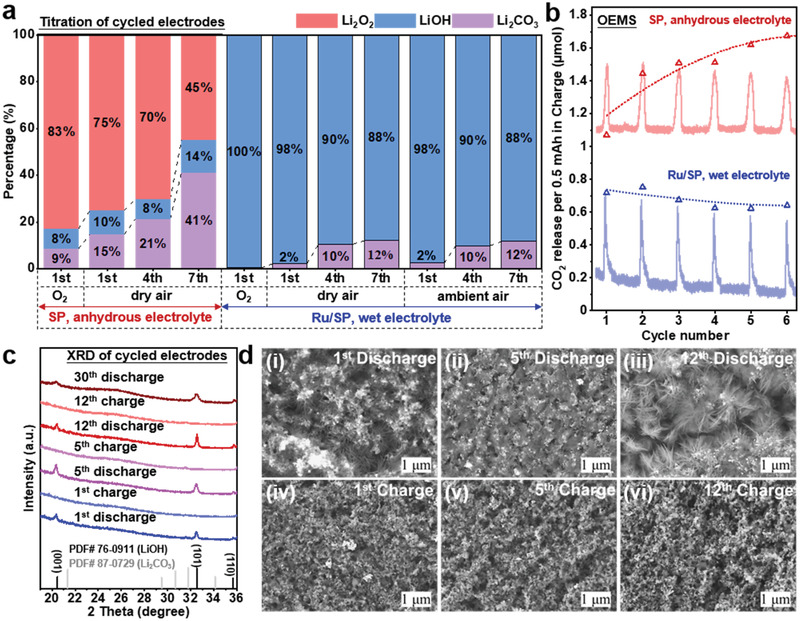
Characterization and quantification of discharge products in Li‐O_2_/air cells over extended cycles. a) Quantification of discharge products in Li‐O_2_/air cell using acid‐base, TiOSO_4_‐based UV–vis spectroscopic and mass spectrometric titrations (see Figures S2–S4, Supporting Information) to quantitatively evaluate Li_2_O_2_ (red bars), LiOH (blue bars), and Li_2_CO_3_ (violet bars) within the cathodes, respectively. The original processed data of quantified discharge products are presented in Table S1 (Supporting Information). b) Moles of CO_2_ release during charge in the first six cycles in Li_2_O_2_‐(SP, anhydrous 1 m LiTFSI/DMSO) and LiOH‐based (Ru/SP, 1 m LiTFSI/DMSO with 5 vol% H_2_O) Li–air cells; the areas of CO_2_ evolution peaks have been integrated and plotted with a trend line. c) XRD patterns and d) SEM images of Ru/SP electrodes at different states of charge.

To provide detailed insights into the reactions occurring at the various voltage plateaus (Figure 2), qualitative and quantitative experiments have been performed over extended cycles, including OEMS, chemical titration, XRD, ssNMR, FTIR and scanning electron microscopy (SEM). OEMS measurements of the first cycle (**Figure** **3**a) revealed that in cells using SP cathodes and anhydrous DMSO electrolytes, the discharge process showed 2.18 electrons per reduced O_2_, consistent with dominant Li_2_O_2_ formation.^[^ ^59^, ^60^, ^61^ ^]^ Chemical titration of the discharged electrodes in dry air (**Figure** **4**a) confirmed that the discharge products are indeed mainly composed of Li_2_O_2_ (75%), together with LiOH (10%) and Li_2_CO_3_ (15%) due to side reactions, the quantities of these by‐products being 2% and 6% higher than those obtained in pure O_2_ (Figure 4a). On charging, around 65.4% of O_2_ was recovered and significant amount of CO_2_ evolved, initiating at ≈4.45 V; the latter process has been mainly attributed to Li_2_CO_3_ decomposition^[^ ^8^, ^11^, ^14^, ^66^, ^67^ ^]^ and associated side reactions, e.g., those involving singlet oxygen.^[^ ^17^ ^]^

For cells with Ru/SP cathodes and wet electrolytes, OEMS showed a discharge process of 3.97 electrons per reduced O_2_ in dry air (Figure 3b), highly consistent with previously reported values in pure O_2_.^[^ ^58^ ^]^ Titrations of discharge products obtained in both dry air and ambient air (Figure 4a) confirmed that the discharge product was overwhelmingly LiOH (98%), very close to that obtained in pure O_2_ (100% LiOH), and that around 2% of the products was Li_2_CO_3_, 7.5 times lower than the Li_2_CO_3_ amount detected in the Li_2_O_2_ case. No Li_2_O_2_ was identified to the detection limit of the titration method. The presence of Li_2_CO_3_ (characteristic absorption bands at 857 and 1400 cm^−1^) was also confirmed by FTIR measurements on discharged samples in air (Figure S7b, Supporting Information).These results suggest that 1) electrochemical LiOH formation in pure O_2_ hardly involves any side reactions, with no trace of Li_2_CO_3_ detected in titration; 2) the influence of dry air and ambient air on LiOH‐based electrochemistry are basically the same during discharge, both leading to a small increase in Li_2_CO_3_ formation, and moisture in air does not alter the reaction product at cathodes; 3) the effect of air is more adverse on the process of Li_2_O_2_ formation than LiOH formation, with the former showing a rise in Li_2_CO_3_ quantity 3 times that of the LiOH case. Upon charging the Ru/SP cells, no oxygen was released (Figure 3b) as observed in previous reports, which has been attributed to the oxygen being trapped as DMSO_2_ in the electrolyte (more discussion later).^[^ ^3^, ^58^, ^68^ ^]^ Some CO_2_ evolution has been recorded, corresponding to the 3.65 V plateau, which is in an agreement with previous assignment of this plateau to Li_2_CO_3_ decomposition.^[^ ^63^, ^69^, ^70^ ^]^ The amount of released CO_2_ in the LiOH case was much less than that in the Li_2_O_2_ case (Figure 3a,b), consistent with the titration results (Figure 4a). Notably, in a pure O_2_ atmosphere LiOH decomposition did not incur CO_2_ evolution on charging to 4 V (Figure 3c), whereas the decomposition of Li_2_O_2_ on charging caused considerable CO_2_ evolution even in pure O_2_; the latter has been linked to charge‐induced side reactions corroding electrolytes and carbon cathodes.^[^ ^11^, ^14^, ^17^, ^66^, ^67^, ^68^ ^]^ This difference implies a better electrochemical stability of LiOH with the carbon cathode and electrolyte.

To further elucidate the long‐term effect of air on the cell electrochemistry, titration, XRD, ssNMR and OEMS measurements have been conducted over several cycles. For cells using SP cathodes and anhydrous electrolytes that were initially a Li_2_O_2_‐dominated electrochemistry in dry air, the discharge products at the fourth and seventh discharge (Figure 4a) showed a substantial increase in the proportions of Li_2_CO_3_ and LiOH, compared to those in the first discharge. Of note, Li_2_O_2_ in the 7^th^ discharge accounts for only 45% in the products, no longer being the dominant electrochemistry; Li_2_CO_3_ formation (41%) becomes the other major process. On the other hand, for cells using Ru/SP cathodes and wet electrolytes, the prevailing electrochemistry during discharge at the fourth (90%) and seventh cycle (≈88%) is still LiOH formation in both dry and ambient air (Figure 4a). The trend of CO_2_ evolution over 6 charge cycles has been analyzed by OEMS (Figure 4b). For cells using SP cathodes, rising CO_2_ evolution signals with cycle number were recorded, whereas cells using Ru/SP cathodes and wet electrolytes revealed relatively stable CO_2_ signals with cycling; these observations are largely consistent with the titration results (Figure 4a) and the trends observed in corresponding charging plateaus (Figure 2c,d), indicating that Li_2_CO_3_ formation during discharge and CO_2_ evolution on charging are closely correlated.

XRD, ssNMR, and SEM characterizations also support that the electrochemistry of Ru/SP cells is predominated by LiOH formation and decomposition over extended cycles in ambient air. Over 30 cycles, the only observable Brag reflections (Figure 4c) at the end of each discharge cycle were all associated with LiOH and they completely vanished after the corresponding recharge. The fact that no Brag reflections of Li_2_CO_3_ were detected in the current study may be linked to its low quantities or amorphous structure. ^7^Li ssNMR measurements on discharged/charged samples over 10 cycles revealed appearance/disappearance of a single resonance situating at 1.1 ppm that is characteristic of LiOH (Figure S7a, Supporting Information). Similarly, SEM images (Figure 4d) illustrate that LiOH formation (flower‐like morphology^[^ ^58^, ^71^ ^]^) and removal were the main processes over 12 discharge–charge cycles, further supporting good rechargeability.

Thus far, it has become clear the prominent air constitute that impacts on the cathode electrochemistry is CO_2_, which tends to cause more Li_2_CO_3_ formation during discharge and more CO_2_ evolution on recharging. The Ru/SP‐catalyzed LiOH electrochemistry is more resistant to this CO_2_‐induced conversion of discharge product and is immune to moisture in air, exhibiting a better electrochemical stability than Li_2_O_2_. Next, we begin to uncover the origins of Li_2_CO_3_ formation and discuss the key factors modulating the CO_2_ effect on the LiOH‐based electrochemistry.

**Figure 5:**
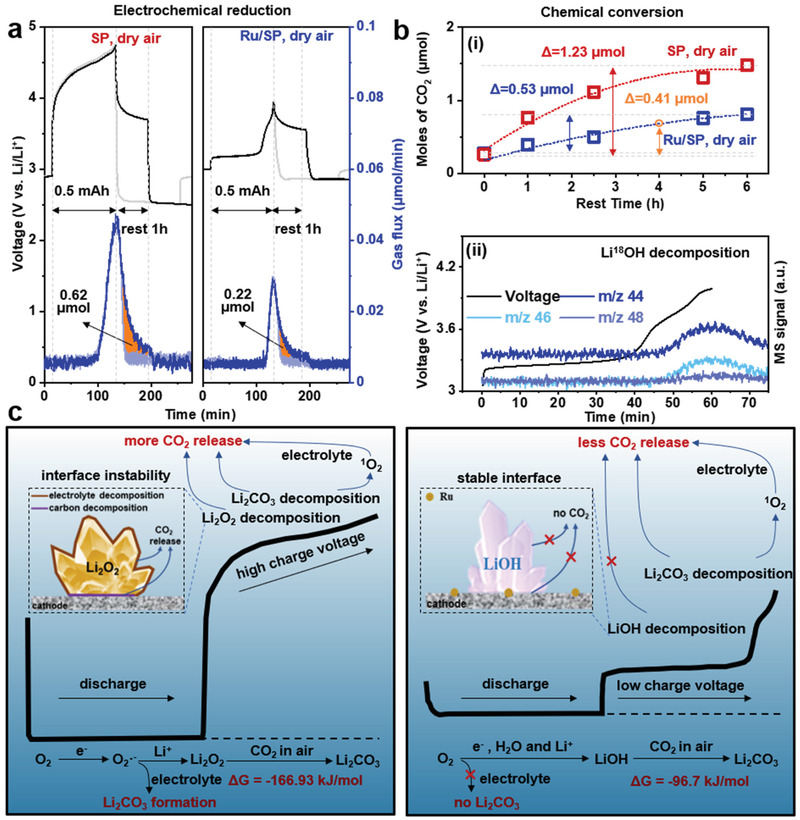
Sources of Li_2_CO_3_ and key factors modulating the cell electrochemistry. a) OEMS showing direct electrochemical reduction of CO_2_ during the subsequent discharge in both Li_2_O_2_‐based (left, SP) and LiOH‐based (right, Ru/SP) Li‐dry air batteries. The amount of CO_2_ electrochemically reduced is shaded by orange color, which is the difference between the CO_2_ peak discharging immediately after the previous charge (dark blue) and that with 1 h resting after charging (light blue). b) Impact of rest time after a discharge on chemical transformation of discharge products and subsequent CO_2_ evolution on charging: i) CO_2_ released on charging Li_2_O_2_‐based and LiOH‐based batteries with varying rest times after a same discharge capacity; ii) Online C^16^O_2_ (*m/z* = 44), C^16^O^18^O (*m/z* = 46), C^18^O_2_ (*m/z* = 48) signals, recorded during electrochemical decomposition of ^18^O‐enriched Li^18^OH. c) A scheme highlighting the sources of Li_2_CO_3_ and the differences between the two systems that could determine the electrochemical stability in air.

The first potential route to be considered is direct electrochemical reduction of CO_2_ to form Li_2_CO_3_ via reaction 2,

(2) $$2{\mathrm{CO}}_{2}+O_{2}+4e^{-}+4{\mathrm{Li}}^{+}\to 2{\mathrm{Li}}_{2}{\mathrm{CO}}_{3}$$

which has been established in Li‐O_2_/CO_2_ batteries employing much higher CO_2_ concentrations (>10%) in the atmosphere.^[^ ^68^, ^72^ ^]^ In the current study, the concentration of CO_2_ for cell reactions used was only 0.038%, the same as in ambient air. Typically, no appreciable drop in the CO_2_ OEMS baseline has been observed during the 1^st^ cell discharge, which however was not the case in subsequent discharges. **Figure** **5**a compares the difference in the decaying signals of CO_2_ in cases with and without a resting period between the prior charge and the following discharge. During a cell charge followed by a resting period (Figure 5a), the CO_2_ signal rose toward the end of charge and decayed exponentially once the cell started to rest. In the SP cells, the case without resting experienced an even sharper drop in CO_2_ signal rather than an exponential decay, the difference (the shaded area) indicating 0.62 µmol more CO_2_ being consumed, whereas in the Ru/SP cells, this quantity of electrochemical reduced CO_2_ following a charge was three times smaller (Figure 5a). In parallel to the process of CO_2_ reduction, the corresponding O_2_ consumption also showed a different behavior: a small step in O_2_ consumption tended to emerge at a level representing four electrons per reduced O_2_ (Figure S8, Supporting Information), which was in response to the distinct discharge plateau at ≈2.8 V, in agreement with the reaction stoichiometry of Equation (2). These observations strongly suggest that electrochemical reduction of CO_2_ is a prominent source for the generated Li_2_CO_3_ during discharge. Of note, the decomposition of Li_2_CO_3_ evolved little O_2_ (Figure 3 and Figure S8, Supporting Information), being highly deviated from the reverse of Equation (2) (that is, irreversible), as also captured in previous studies.^[^ ^68^, ^69^, ^70^, ^72^ ^]^

Unlike the flow‐mode in OEMS measurements, a typical lithium–air cell operates in the absence of dynamic gas flow, so that CO_2_ released during charges dissipates much slower, creating a CO_2_‐enriched environment especially at the electrolyte‐electrode interface. It is clear that in the SP cells, these previously released CO_2_ during charge could be reduced and fixed as Li_2_CO_3_ again (Figure S8a, Supporting Information), which was in turn decomposed in the next charge, causing additional CO_2_ evolution. This cumulative effect on Li_2_CO_3_ formation and decomposition likely led to elongation of the Li_2_CO_3_ formation plateau at 2.81 V (Figure 2c) and the rising and longer Li_2_CO_3_ decomposition voltages on recharging (Figure 2c and Figure S6a, Supporting Information). Eventually, a Li_2_O_2_‐dominated electrochemistry has been converted to a Li_2_CO_3_‐dominated process. No such cumulative effect of Li_2_CO_3_, however, was reflected in the Ru/SP‐catalyzed LiOH electrochemistry, the electrochemical profile (Figure 2d), the proportion of the Li_2_CO_3_ decomposition plateau (Figure 2d), the titrated quantities of Li_2_CO_3_ in discharge products (Figure 4a) and the moles of CO_2_ released in each charge (Figure 4b) all being relatively stable over extended cycles.

Another potential route to Li_2_CO_3_ formation is via chemical conversion. The exposure time, i.e., resting period between discharge and the following charge, of reaction products to a dry air flow has been varied between 0 and 6 h (Figure S9, Supporting Information). The resulting CO_2_ evolution peaks during the following charge have been integrated and plotted in Figure 5b, which showed that the rise in CO_2_ evolution grew faster in the Li_2_O_2_ case than in the LiOH case, eventually being ≈2.5 times higher at 6 h resting; this observation implies that LiOH is more chemical resistance to CO_2_ contamination than Li_2_O_2_, consistent with the thermodynamic consideration (Figure 5c).^[^ ^3^ ^]^ To further verify that chemical conversion of LiOH by CO_2_ to form Li_2_CO_3_ is a valid route to the released CO_2_ on recharging, Li^18^OH was first generated during discharge using ^18^O‐enriched H_2_^18^O and then charged in dry air; the *m/z* signals of C^16^O_2_, C^16^O^18^O, C^18^O_2_ in the resulting OEMS data all show a peak. Given that half of the oxygen in LiOH originates from H_2_^18^O^[^ ^49^, ^58^ ^]^ and that electrochemical LiOH formation in O_2_ does not involve side reactions generating Li_2_CO_3_ (Figure 4a), nor does CO_2_ evolution occurs during electrochemical LiOH decomposition (Figure 3c), it is hence sensible that the above isotopic scrambling of CO_2_ necessitates incorporation of ^18^O in Li_2_CO_3_ via reacting CO_2_ with Li^18^OH.

For a capacity of 0.5 mAh per cycle in a total duration of 4 h (Figure 4b), the average CO_2_ evolution due to Li_2_CO_3_ decomposition in each charge was ≈0.67 µmol over 6 cycles, only 32.8% of which (0.22 µmol) was electrochemically reduced again (Figure 5a), the rest of Li_2_CO_3_ being expected to come from other sources. Of note, for a period of 4 h exposure to air, the rise in Li_2_CO_3_ in the Ru/SP cells due to chemical conversion approximated to 0.41 µmol (orange arrows, Figure 5b(i)), which could roughly be responsible for the rest of CO_2_ released. Therefore, the chemical conversion of LiOH to Li_2_CO_3_ by CO_2_ in air appears to account for a larger fraction of the Li_2_CO_3_ formed in each cycle, although electrochemical reduction of CO_2_ cannot be completely ruled out for the rest of the discharge capacity when the CO_2_ concentration in the electrolyte is equilibrated with the atmosphere.^[^ ^65^, ^73^ ^]^

Li_2_CO_3_ formation/decomposition increases charge overpotentials and corrodes the cathode and electrolyte, it is therefore worth discovering effective methodologies to minimize its fraction in the electrochemistry. Three factors (Figure S10, Supporting Information) have been identified affecting the plateau (3.65 V) proportional to Li_2_CO_3_ formation/decomposition, i.e., water concentration in electrolytes, cycling rates, and the discharge depth. Higher water concentrations (up to 5 vol.%), faster cycling rates, and deeper discharge capacities tended to lead to smaller fractions of Li_2_CO_3_.

As illustrated in Figures S6 and S10a (Supporting Information), at smaller water concentrations the cell exhibited a mixed electrochemistry of Li_2_O_2_ and LiOH, the former being more vulnerable to CO_2_ contamination, thus forming more Li_2_CO_3_. As the water concentration was increased (Figure S10a, Supporting Information), the cell reaction resembled more of LiOH formation, and hence becoming more resistant to CO_2_ in air; this may be one aspect to account for the observed water effect modulating the cell electrochemistry. As for faster cycling rates, it in effect shortened the exposure time of discharge product in air for chemical conversion (Figure S10b, Supporting Information). Finally, deeper discharge capacities involved formation of larger LiOH particles, which tended to have a smaller specific surface area per capacity for chemical reactions with CO_2_; this may account for the smaller proportion of 3.65 V plateau in the whole charge capacity (Figure S10c, Supporting Information).

Taken together, the Ru/SP‐catalyzed LiOH electrochemistry has clearly demonstrated a superior stability over the Li_2_O_2_‐electrochemistry, as reflected in the overlapping voltage profiles over extended cycles (Figure 2), in the similar quantities of Li_2_CO_3_ titrated at different discharges (Figure 4a), and in the stable CO_2_ evolution peaks over many charge cycles (Figure 4b). This superiority in electrochemical stability is proposed to be rooted in the intrinsically more stoichiometric electrochemistry of LiOH compared to Li_2_O_2_: lack of Li_2_CO_3_ generation during electrochemical LiOH formation, the process of LiOH decomposition incurring no CO_2_ evolution, a relatively stable LiOH‐carbon interface, and a better chemical resistance to CO_2_, which, however, are all known issues plaguing the Li_2_O_2_ electrochemistry, as schematically depicted in Figure 5c.^[^ ^8^, ^11^, ^14^, ^66^, ^67^ ^]^

**Figure 6:**
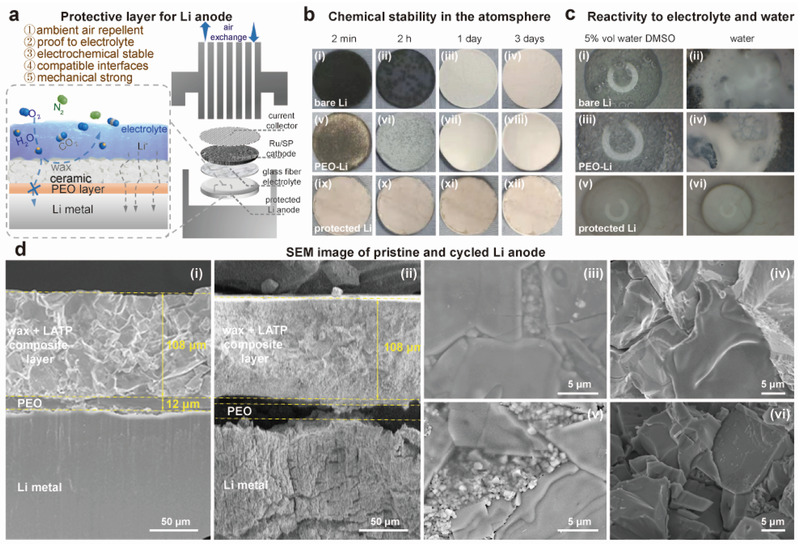
Scheme illustrating the design strategy of a nonporous protective layer on Li metal and demonstration of its excellent stability in air, water, and in an open lithium air battery. a) Cartoon illustrating the design of a multifunctional composite layer on Li anodes and components in a typical cell based on a Swagelok design. b) Optical images showing i–iv) bare Li, v–viii) PEO‐coated Lim and ix–xii) composite‐protected Li exposed directly in atmosphere for 2 min, 2 h, 1 d, and 3 d. c) Optical snap shots showing reactivities of i,ii) bare Li, iii,iv) PEO‐coated Li, and v,vi) composite–protected Li with wet 1 m LiTFSI/DMSO electrolytes (5% H_2_O) and pure water. d) SEM images of pristine Li anodes i,iii,iv) protected by the composite layer, ii, v, vi) protected Li anodes after being subjected to around 100 electrochemical cycles.

In the following part, we turn to discussing lithium anode protection and its application in a full lithium–air battery (**Figure** **6**). Preparing an air‐stable lithium metal anode is essential to simplification and cost reduction of the manufacturing process for practical lithium anodes,^[^ ^33^, ^34^, ^40^, ^74^, ^75^, ^76^, ^77^, ^78^ ^]^ and is a prerequisite to realization of a long‐life open lithium–air battery.^[^ ^2^, ^3^ ^]^ When exposed in air, a piece of bare lithium metal immediately reacted with the constitutes of air (Figure 6b), its appearance turning from metalescent to black within a minute, then to a grey color and eventually to completely white in a couple of days. XRD measurements showed that LiOH was the dominant reaction product formed on the Li disc (Figure S11, Supporting Information), implying that moisture in air is the major threat to a bare Li anode.^[^ ^7^, ^79^ ^]^ Furthermore, the electrolyte used in the current study was LiTFSI/DMSO with a substantial water content (5 vol%), where it is known that DMSO is unable to form a stable SEI on lithium^[^ ^59^, ^80^, ^81^ ^]^ and water reacts violently with lithium releasing heat and H_2_ gas (Figure 6c), both representing additional challenges to achieve an electrochemical stability for Li anodes. Similar to many previous studies,^[^ ^23^, ^24^, ^25^, ^30^ ^]^ we initially addressed the issues by implementing a dual compartmental design in a full cell by the use of a piece of Ohara glass disc (Figure S12a, Supporting Information), which separated the water‐containing catholyte from an anhydrous ether‐based anolyte. In dry air, this full cell design exhibited electrochemical performance (Figure S12b, Supporting Information) similar to that of a half cell (Figure 2), and OEMS confirmed the discharge reaction of the full cell had close to four electrons per reduced O_2_ (Figure S12c, Supporting Information), consistent with LiOH formation. Nonetheless, the lithium anode could not survive in real air conditions using this dual compartmental design. The cell failed after two days of cycling test, because of lithium corrosion by moisture leaking through the gaps at the peripheral regions of this cell. Therefore, a layer that could more uniformly and sufficiently protect Li anodes is required.

To ensure a stable electrochemical performance of a lithium anode that is coupled to a LiOH‐based cathode in air, the protective layer on lithium needs to have at least the following properties: 1) repelling N_2_, H_2_O, CO_2_, O_2_ from air so that Li anodes can be air‐stable in the long term; 2) proof to liquid electrolyte so that the protected Li anodes can be compatible with a wide range of nonaqueous electrolytes, even those with high water contents; 3) supporting a high Li^+^ conductivity; 4) possessing a stable interface between lithium metal and the protective layer; 5) having a sufficient mechanical strength so that Li dendrite formation is suppressed. Given the above consideration, we have constructed a nonporous composite protective layer (Figure 6a), which is composed of a thin PEO film (≈10 µm) coated directly on Li metal and then on top of the PEO film, a composite layer (≈100 µm) of sintered LATP layer infiltrated with wax, as shown in Figure 6d(i,iv). The bare LATP surfaces facing electrolytes and the wax within the gaps of LATP grains can be observed in Figure 6d(iii,iv).

The designing principles are explained below. The soft and flexible PEO layer could provide a chemically stable interface with Li metal^[^ ^82^ ^]^ and a gapless contact with the LATP layer for Li^+^ conduction.^[^ ^38^, ^83^, ^84^, ^85^ ^]^ The nonporous, mechanically strong LATP‐wax composite layer is designed to protect the rest of the Li anode from air and reactive species in electrolytes, as well as help suppress Li dendrite growth. Ideally, the thickness of the all‐solid‐state composite layer should be made thinner (<50 µm)^[^ ^86^ ^]^ in order reduce resistance for charge transfer and improve the rate capacity of the lithium anode. Conventional polymers containing polar segments such as PEO, PAA (Polyacrylic acid), cannot replace the role of wax, because these polymers are microporous and permeable to water vapor and electrolyte solvents. During cell operation, components of air and liquid electrolytes are not allowed to get across this protective layer (Figure 6a), but Li^+^ could diffuse through the sintered grains of LATP and then the PEO film. It is feasible that driven by the potential difference in the cell, directional diffusion lithium ions occur in LATP layer toward the lithium anode, creating Li^+^ vacancies at the LATP‐liquid electrolyte interface. Desolvation of Li^+^ may happen preferentially at these vacancy sites and then insert into the Li^+^ diffusion channels in LATP.^[^ ^78^, ^79^, ^80^, ^81^, ^82^, ^83^, ^84^, ^85^, ^86^, ^87^, ^88^ ^]^ EIS measurements on the protective composite layer with/without wax showed that wax did not induced additional resistance to Li^+^ transport through the protective layer (Figure S13, Supporting Information).

To examine its chemical resistance to air and water, protected Li anodes by the composite layer have been exposed in air and to water (Figure 6b,c). No observable changes occurred after 3 days to the Li anode in air, nor was gas bubbling seen when it was in direct contact with the wet electrolyte or water (Figure 6b,c and Movie S1, Supporting Information); these observations strongly support that the protected Li anode is air‐stable and liquid‐proof. When lithium metal was only covered by a PEO layer, rapid corrosion by air and water was observed (Figure 6b,c and Movie S1, Supporting Information), suggesting that PEO being a microporous film is unable to effectively filter aggressive gas molecules,^[^ ^7^, ^75^ ^],^ e.g., H_2_O and CO_2_. Of note, wax is also essential to achieving adequate protection. When in a liquid form at 65˚C, wax effectively wet and filled up the microcracks of the LATP layer. Without wax filling up the gaps, reactive molecules and electrolytes readily leaked through and caused corrosion (Movie S1, Supporting Information).

**Figure 7:**
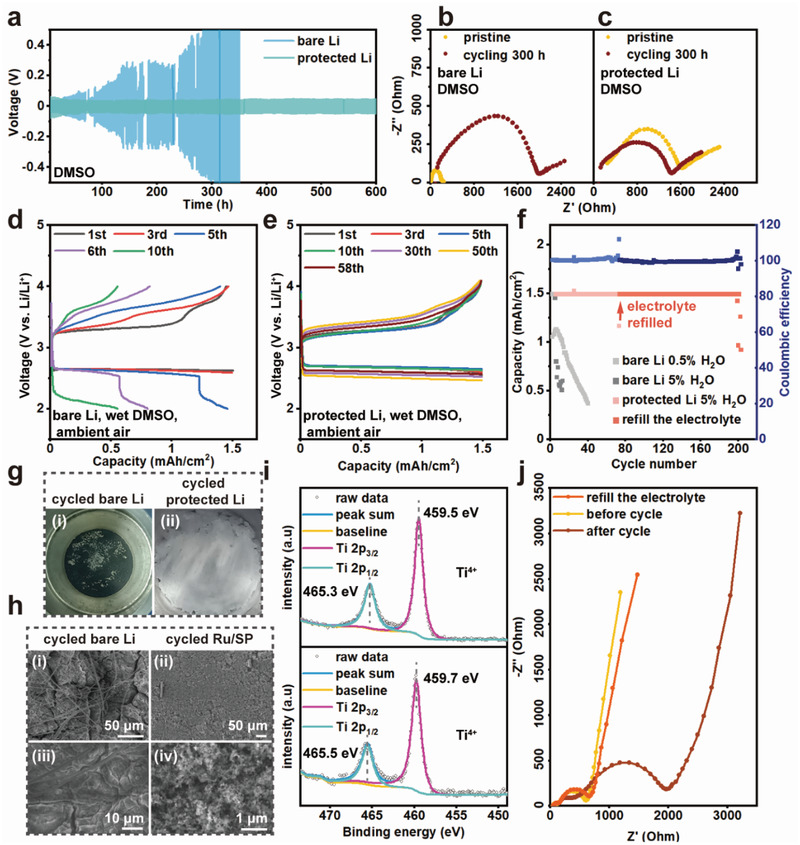
Electrochemical performance of full lithium–air batteries using protected lithium anodes. a) Electrochemical profiles of Li/Li symmetric cells using protected lithium and bare lithium, at 0.015 mA and 0.15 mA, respectively. b) EIS spectra acquired at room temperature on cells in (a) after 300 h cycling. d,e) Electrochemical performance of lithium–air full cells in ambient air using 1 m LiTFSI/DMSO with 5 vol% H_2_O. f) Cycling stability of lithium–air full cells in (d) and (e). g) Optical image of i) cycled bare Li and ii) protected Li anodes extracted from full cells in (d) and (e). h) SEM images of the i,iii) cycled bare lithium anode from g‐i) and ii,iv) a Ru/SP cathode at the end of cycle life (at conditions as in (e)). i) Ti 2p XPS spectra acquired on the surfaces of a protected lithium anode (bottom) after cycling and a pristine LATP plate (top). j) EIS of a lithium–air full cell (using a protected Li anode) at different stages of its cycle life.

To further evaluate the electrochemical stability of the protected lithium anodes, galvanostatic cycling has been tested on Li/Li symmetric cells using the DMSO electrolyte, which is known to be incompatible with bare lithium anodes.^[^ ^59^, ^89^, ^90^ ^]^ The electrochemical profile of protected lithium anodes was clearly more stable than that of bare lithium, with little increases in polarization over 600 h (**Figure** **7**a). Electrochemical impedance spectra showed that the charge transfer resistance of bare lithium anodes had increased by ≈8 times (Figure 7b) after 300 h cycling, whereas that of the protected lithium anodes remained roughly the same after an equivalent testing (Figure 7c).

Moreover, full lithium–air cells using the protected lithium were cycled in ambient air (55% humidity), and compared to results obtained using bare lithium as anodes. Without Li protection, the full cell using wet electrolytes (5% and 0.5% H_2_O) in air started to show severe capacity fade and larger polarization after just 5 cycles (Figure 7d,f). The charging profiles gradually reverted to that of cells using an anhydrous electrolyte, which is probably due to consumption of water by the lithium anode (Figure 6b,c). The appearance of the lithium anode became black (Figure 7g(i)) and highly corroded (Figure 7h(i,iii)) after 10 cycles. On the contrary, the cells using the composite‐protected lithium anodes exhibited an excellent electrochemical stability (Figure 7e,f) over extended cycles in the wet electrolytes (5% H_2_O), with one discharge and two charge plateaus at 2.7, 3.2 and 3.65 V, respectively; the electrochemical profiles were similar to those observed in half cells where lithium iron phosphate was used as the counter electrode (Figure 2d). We have even cycled the full lithium–air battery in air at maximum 99% humidity, and the electrochemistry of these cells showed no difference from the same cells operating in air with a lower humidity (Figure S14, Supporting Information).

XPS measurements (Figure 7i) on a PEO‐LATP‐wax protective layer from a full cell subjected to 100 cycles in air showed no obvious change in the Ti^4+^ oxidation state (Ti 2p_3/2_ and Ti 2p_1/2_) of LATP surfaces, suggesting that LATP was not reduced by lithium dendrite. The appearance of protected Li anodes after cycling remained white (Figure 7g(ii)), unlike the color for corroded LATP layer by lithium (Figure S15, Supporting Information), and no lithium dendrite either growing on top of the protective layer or penetrating along the grains of the LATP composite layer had been found by SEM (Figure 6d(v,vi) and Figure S16, Supporting Information). Underneath the PEO layer (Figure 6d(ii)), mossy structures (compact porous microstructures) of lithium have been observed, which again confirms that Li^+^ transported across the protective layer and deposited at the lithium‐PEO interface. The absence of dendrite penetrating across the protective layer is also supported by a constant fraction of the 3.65 V plateau over many cycles, which indicates that the content of water in the electrolyte was not consumed by lithium during electrochemical plating and stripping. The suppression of dendrite growth is tentatively ascribed to the high mechanical strength of the LATP‐wax composite layer that is pressed onto the PEO‐coated lithium anode.^[^ ^37^, ^75^, ^91^ ^]^ In the absence of the LATP component, however, lithium anodes protected only by a PEO‐wax layer showed considerable lithium dendrites penetrating through the protective layer (likely through the soft PEO component)^[^ ^34^ ^]^ and growing on top of the surface (Figure S17, Supporting Information); this dendrite growth in turn consumed water in the electrolyte and converted the cell reaction toward a Li_2_O_2_‐dominated electrochemistry, as supported OEMS (Figure S18, Supporting Information).

Typically, a full lithium–air battery could cycle in air for ≈100 cycles at a capacity of 1.5 mAh cm^−2^ (Figure 7f) before capacity fades, similar to that of half cells (Figure 2f). The sum of ohmic and charge transfer resistances of a failed cell was observed to increase to over 2000 ohms (Figure 7j), compared to a value of ≈600 ohms prior to long‐term cycling, and the glass fiber separator of the cell had become completely dried. ^1^H and ^13^C solution NMR measurements (Figure S19, Supporting Information) of the cycled separator showed that considerable DMSO_2_ had been generated after cycling (more discussion on cell failure in Figure S19a,b, Supporting Information),^[^ ^58^ ^]^ whereas C 1s and Ru 3p XPS spectra of the cycled electrode (Figure S19c,d, Supporting Information) remained largely the same as those for pristine Ru/SP electrodes, consistent with the absence of solid by‐products extensively covering the cycled cathode (Figure 7f(ii,iv)). Taken together, these results imply that that the cell failure is likely connected to depletion of the electrolyte, i.e., DMSO being oxidized to DMSO_2_, rather than failure of the protected Li anode or the Ru/SP cathode. After the glass fiber separator was replaced by a new one filled with a fresh electrolyte, the impedance of that cell dropped to ≈600 ohms (Figure 7j) and it can cycle at 1.5 mAh cm^−2^ again for ≈100 cycles (Figure 7f), further demonstrating that the protected Li anode was not the bottle‐neck to cycle life and it possesses a high electrochemical stability. It is anticipated that when a fully reversible LiOH‐based electrochemistry is applied at the cathode, the rechargeability of the full cell in air can be further improved. Tables S3 and S4 (Supporting Information) have been compiled to make a clear comparison of the operational conditions and the performance of batteries in this work with relevant studies in literature. As can be seen, no prior study has achieved such an excellent performance under the harsh conditions demonstrated in the current work. In terms of capacity and cycle life of full lithium–air batteries, our results are also competitive to many of the best work reported.

In conclusion, we have shown that the most prominent constitute in air that affects the O_2_ electrochemistry is CO_2_, which accelerates the transformation of an initial Li_2_O_2_‐based electrochemistry to a Li_2_CO_3_‐dominated process during battery cycling. On the contrary, the Ru‐catalyzed LiOH electrochemistry shows much better resistance to CO_2_ contamination in air, the nature of the cell reaction being consistent over extended cycles. Compared to the sensitivity of Li_2_O_2_ to air, we ascribe this superior stability of the LiOH electrochemistry to the higher chemical resistance of LiOH, a stoichiometric process of LiOH formation, less reactive product‐cathode interfaces, and lower charge voltages. We have also constructed an all‐solid‐state protective layer consisting of PEO, LATP and wax, which has rendered lithium metal chemically stable in air and pure water for a long term, fulfilling an essential prerequisite to the realization of a highly rechargeable and energy‐dense lithium–air battery. As a proof of concept, our results demonstrate that this strategy of lithium protection enables reversible, dendrite‐free lithium plating/stripping under a combination of aggressive operating conditions, including in ambient air, at up to 99% humidity, and even in lithium‐incompatible electrolytes (DMSO) with substantial amount of water. The current approach of coupling a protected lithium anode with a LiOH‐based cathode holds promise for developing a long‐life, high‐energy lithium–air battery in the ambient atmosphere.

## Conflict of Interest

The authors declare no conflict of interest.

## Supporting information

Supporting Information

Supporting Information

## Data Availability

Research data are not shared.
