# Supplementary material for: Coupling Water‐Proof Li Anodes with LiOH‐Based Cathodes Enables Highly Rechargeable Lithium–Air Batteries Operating in Ambient Air
Source: Adv Sci (Weinh). 2021 Dec 11;9(4):2103760. doi: 10.1002/advs.202103760 (PMC8811808; doi:10.1002/advs.202103760)
Supplement: Supplementary file 1 — Supporting Information [file ADVS-9-2103760-s001.pdf]

ADVANCED SCIENCE
Open Access

Supporting Information

for *Adv. Sci.*, DOI: 10.1002/advs.202103760

Coupling Water-Proof Li Anodes with LiOH-based Cathodes Enables Highly Rechargeable Lithium-Air Batteries Operating in Real Air

Jiang Lei,[1,#] Zongyan Gao,[1,#] Linbin Tang,[1] Li Zhong,[2,] Junjian Li,[1] Yue Zhang,[1] Tao Liu[1,*]*

Coupling Water-Proof Li Anodes with LiOH-based Cathodes Enables Highly Rechargeable Lithium-Air Batteries Operating in Real Air

Author Names

Jiang Lei,[1,#] Zongyan Gao,[1,#] Linbin Tang,[1] Li Zhong,[2,*] Junjian Li,[1] Yue Zhang,[1] Tao Liu[1,*]

Institutes

[1] Shanghai Key Laboratory of Chemical Assessment and Sustainability, School of Chemical Science and Engineering, Tongji University, No. 1239, Siping Road, Shanghai, 200092 P. R. China

[2] SEU-FEI Nano-Pico Center, Key Laboratory of MEMS of Ministry of Education, Southeast University, Nanjing 210096, China.

* Correspondence to: tliu@tongji.edu.cn, and lizhong@seu.edu.cn

Supporting Information

Contents

1. Materials and Methods 3
1.1. Chemical and reagents 3
1.2. Preparation of Ru/SP 3
1.3. Preparation of electrodes 4
1.4. Preparation of PEO-LATP-Wax protective layers 4
1.5. Cell assembly and electrochemical measurements 5
1.6. Scanning electron microscopy (SEM) and transmission electron microscopy (TEM) 6
1.7. X-ray diffraction (XRD) 7
1.8. X-ray photoelectron spectroscopy (XPS) 7
1.9. Nuclear magnetic resonance spectroscopy (NMR) 7
1.10. Operando Electrochemical Mass spectrometry (OEMS) 8
1.11. Fourier transform infrared spectroscopy (FTIR) 9
2. Quantification of the Discharge Products 10
2.1. Preparation of discharged cathodes 10
2.2. Li_2CO_3 calibration 10
2.3. Li_2O_2 calibration 11
2.4. LiOH calibration 12
2.5. The original processed data of quantified discharge products 13
2.6. Evaluation of measurement errors in titration 14
3. Characterization of Ru/SP 15
4. Li-dry air battery using Ru/SP electrodes with anhydrous electrolytes 16
6. CO_2 accumulation impact on Li_2O_2-based electrochemistry 20
7. The impact of resting time on CO_2 evolution during recharging 22
8. Factors affecting the fraction of Li_2CO_3 formation 24
9. XRD characterization for Li metal exposed in ambient air 25
10. Compartmental Li-air full cell using Ohara ceramic glass 26
12. Protection strategy of Li anodes 28
13. Characterization for cycled electrolyte and air cathode 32
14. Tables comparing our work with others in literature 33
15. Reference 36

1. Materials and Methods

1.1. Chemical and reagents

Dimethyl sulfoxide (DMSO, >99.9%, Macklin) and tetraethylene glycol dimethylether (TEGDME, ≥99.9%, Dow Chemical) were dried over freshly activated 4 Å molecular sieves for weeks. Lithium bis(trifluoromethanesulfonyl)imide (LiTFSI, purity of >99.99%, Solvay) was used as the lithium salt after drying in vacuo at 160°C for 24 h. All chemicals were stored in an Ar-filled glove box (Mikrouna) with both H_2O and O_2 concentrations lower than 0.01 ppm before use. Electrolytes of 1 M LiTFSI in DMSO or TEGDME were prepared and stored in a glove box under an Ar atmosphere. Various added water contents were based on volume percentage. For electrolyte with ^{18}O-enriched water, ^{18}O-enriched water (with enrichment levels of 97% $H_2{}^{18}O$ and 3% $H_2{}^{16}O$, Shanghai Maotu Gases Co., Ltd.) was used as purchased. Oxygen (O_2, 99.995%, Shanghai ChunYu Special Gases Co., Ltd), argon (Ar, 99.999%, Shanghai ChunYu Special Gases Co., Ltd) and dry air (21.2% O_2, 0.038% CO_2, 78.76% N_2, Shanghai ChunYu Special Gases Co., Ltd) were employed directly without any further purification.

1.2. Preparation of Ru/SP

Procedures were based on our previous work.[1] Briefly, 300 mg of Super P (SP, Timcal) was dispersed in 100 ml of ethanol (EtOH, ≥99.7%, Macklin) containing 46.6 mg of $RuCl_3$ (purity of 99.99% metals basis, Macklin), and stirred continuously until the solution evaporated completely. The resulting mixture was subsequently reduced in a 5% H_2/Ar mixture atmosphere at 230°C for 2 hours. Then, the final product Ru/SP was collected.

1.3. Preparation of electrodes

The cathode film composed of Super P and polytetrafluoroethylene (PTFE, a dispersion of 60wt%, Macklin) with a ratio of 80:20wt% was kneaded and rolled into a thin film. The film was subsequently punched out to form free-standing electrodes of various sizes (0.5-2 cm^2) and further dried in vacuo at 160°C for 12 hours before the battery assembly. Typically, the areal loading of the electrode is around 1 mg/cm^2. For preparation of Ru/SP cathodes (SP/Ru/PTFE=75:5:20wt%) and lithium iron phosphate/ferric phosphate electrodes (LFP (Canrd)/FP (Aladdin)/SP/PTFE = 40:20:20:20wt%), a similar procedure was conducted.

1.4. Preparation of PEO-LATP-Wax protective layers

25 mg of PEO (Polyethylene oxide) (average Mv ~600,000, Aladdin) were added to 1 ml of TEGDME (tetraethyleneglycol dimethyl ether) and stirred for 6 h at 80°C until a clear solution was obtained. Lithium sheets (diameter, 15.6 mm) were held by a flat tweezer and immersed in the solution for seconds. Then, lithium sheets were placed on a hot plate for 24 hours and then transferred into vacuum overnight to remove the excessive solvent. After drying, PEO-coated lithium metal anodes were obtained. LATP (lithium aluminum titanium phosphate) powder (~300 nm in diameter, Shenzhen, Kejing) was pressed onto mirror-polished stainless-steel sheets under a pressure of around 12 MPa and sintered at 850 °C for 10 h at a heating rate of 3 °C min^{-1} in air. Subsequently the LATP films were transferred into Ar filled glove box and pressed onto the Li-PEO at 60°C. When pressed, the soft PEO layer conforms to provide a good interface with LATP and it also avoids LATP degradation if in direct contact with Li metal. Liquified wax was then infiltrated into the porous LATP film at 65°C, so that the microcracks of LATP were filled up. After cooling, the multifunctional protective layer has been fabricated. The areal loading of the composite protective layer is estimated to be ~5.6 mg/cm^2.

Preparation of a PEO-wax layer on Li metal has been reported previously.[2] Briefly, in our case, 25 mg of PEO (average Mv ~600,000, Aladdin) and 100 mg of wax (paraffin wax, Damas-beta) were added to 1 ml of toluene and stirred for 6 h at 80℃ until a clear solution was obtained. Lithium sheets (15.6 mm in diameter, 0.6 mm or 0.15 mm in thickness) were immersed in the solution for seconds. Then, lithium sheets were placed on a hot plate for hours and then transferred into vacuum overnight to remove the excessive solvent. After drying, PEO-wax-coated lithium metal anodes were obtained.

1.5. Cell assembly and electrochemical measurements

All batteries were assembled using a Swagelok design in a glove box. A typical half-cell was assembled by successively stacking the LFP/FP counter electrode (diameter, 22 mm), a piece of glassy microfiber filter paper (GF/A, Whatman) with around 100 µL of the electrolyte, a SP or Ru/SP cathode and finally a stainless-steel mesh as the current collector (open area of 33%). To fabricate a full cell, a Li anode with the protective layer, a piece of glassy microfiber filter paper (GF/A, Whatman) with around 100 µL of the electrolyte, a Ru/SP cathode and finally a stainless-steel mesh as the current collector were stacked in sequence. In the two-compartment full cells, the following items were stacked in sequence: a lithium sheet as the anode, a piece of glassy microfiber filter paper wetted with 1 M LiTFSI/TEGDME (anolyte), a disk of lithium ion conducting glass ceramic (LICGC, Ohara) membrane preventing the anode from the influence of water and/or carbon dioxide contamination, a piece of glassy microfiber filter paper wetted with 1 M LiTFSI/DMSO with 5 vol% added water (catholyte), and finally the Ru/SP cathode.

Before each galvanostatic test, the cells were rested for 30 min. For all batteries, galvanostatic electrochemical measurements were carried out using the battery testing system CT-4000

(Neware) at room temperature. All potentials in this study were referenced to Li/Li^+. After the test, the electrode was extracted from the cell and rinsed by dry acetonitrile (< 1 ppm H_2O) several times (each time with 2 ml for 10 minutes), and dried under vacuum for further characterization. Electrochemical impedance spectroscopy (EIS) has been performed using an electrochemical workstation (Ivium, Vertex.C.EIS). Electrochemical impedance spectra were acquired at open circuit voltage by applying a sinusoidal wave with an amplitude of 10 mV in a frequency range from 0.01 to 1,000,000 Hz.

1.6. Scanning electron microscopy (SEM) and transmission electron microscopy (TEM)

Morphologies of various cathodes were acquired by a Hitachi-S4800 scanning electronic microscopy (SEM) and a JEOL-2010 high-resolution transmission electron microscopy (TEM), both equipped with energy-dispersive spectroscopy (EDS). The dried cathodes were taken to the SEM sample loading chamber in a sealed sample holder to minimize air exposure. The time from opening the sealed sample holder to finishing sample loading into electron microscopes was < 10 seconds.

The Ru/SP powder was dispersed in ethanol, and it was sonicated for 10 minutes to obtain the well dispersed solution. Then, the solution was dropped onto a Cu grid pre-coated with the Lacey carbon and excessive solvent was removed in vacuum overnight.

1.7. X-ray diffraction (XRD)

XRD measurements were performed using a DX-2700B powder diffractometer (Dandong Haoyuan Instrument Co., Ltd.) operated at 40 kV and 30 mA with Cu Kα as the irradiation source (λ = 1.5405 Å). The dried electrode was put into a home-made X-ray sample holder that was sealed with Kapton polyimide films. XRD is employed to investigate the samples in the 2θ range of 19–39°, using a step size of 0.02° and a scan rate of 0.4° per minute.

1.8. X-ray photoelectron spectroscopy (XPS)

XPS measurements were conducted on a Kratos AXIS-HS spectrometer equipped with a monochromatic Al Kα X-ray source (150 W, 15 kV, 10 mA). The pass energy for the fixed analyzer transmission mode is 40 eV, and the scanning spot size is 20 μm × 20 μm, with a step size of 5 μm. C 1s, Ru 3p and Ti 2p spectra were acquired at a step size of 0.1 eV/sec over 270-300 eV, 446-498 eV and 448-479 eV, respectively.

1.9. Nuclear magnetic resonance spectroscopy (NMR)

Solution ^{1}H-NMR and ^{13}C-NMR spectra were acquired using an ADVANCE III HD spectrometer (400 MHz), chemical shifts are quoted in ppm referenced to an appropriate reference solvent peak. Typically, the glass fiber separator after battery cycling was immersed into DMSO-d_6 (0.7 ml, 99.8%, Acros) solvent for 15 minutes. The resulting solution was transferred into an NMR tube and then the tube was sealed with a cap and parafilm.

A rotor (3.2 mm) synchronized Hahn-echo pulse sequence was used to acquire ^{7}Li magic angle spinning (MAS) spectra with a spinning speed of 55-60 kHz, with a recycle delay of 20 s, and an RF field strength of 125-170 kHz. ^{7}Li shifts was externally referenced to Li_2CO_3 at 0 ppm.

1.10. Operando Electrochemical Mass spectrometry (OEMS)

A newly designed OEMS system was described in Figure S1. The OEMS system consists a 1/16” polypropylene tube carrying a working gas from an electrochemical cell to a mass spectrometer (QMG250M1, Pfeiffer, Linglu Instrument Co., Ltd). The cell design consists in a 1” Swagelok-type cell with inlet and outlet tubes (1/16”) welded to the top plunger. The entire system was hermetically sealed. The mass spectrometer was calibrated to determine the partial pressure of a standard mixture of 500 ppm O_2 and 5000 ppm CO_2 in Ar. The flow velocity of the working gas (21.2% O_2, 0.038% CO_2 and 78.76% N_2) and carrier gas (Ar, ≥99.999%) was controlled at 0.09 ml/min and 2 ml/min via two mass flow controllers (Bronkhorst), respectively. Before in situ DEMS characterization, the residual gas in the cell was purged by Ar until stable O_2 and CO_2 partial pressures were obtained, and the OEMS cells were cycled using a battery tester (CT-4000, Neware).

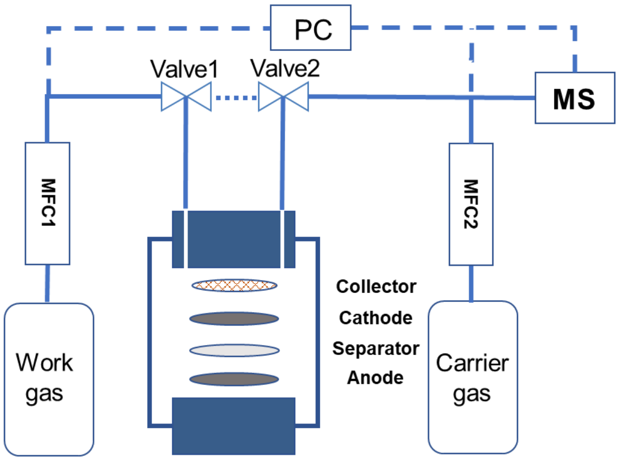

Figure S1. Schematic illustration of the operando electrochemical mass spectrometry system used in this work.

Working gas was controlled via mass flow controller 1 (MFC1), then work gas through the electrochemical cell and further mixed with carrier gas (controlled via mass flow controller 2, MFC2) to mass spectrometer (MS). Broken lines represented signal controls of MFC1, MFC2 and MS via personal computer (PC), and solid lines represented polypropylene tube carring gas.

The dashed line between valves 1 and 2 represents a tube, which will be connected when the battery is not in operation, so as to prevent air pollution to the OEMS system.

1.11. Fourier transform infrared spectroscopy (FTIR)

Fourier-transform infrared spectra were recorded on a Bruker ALPHA spectrophotometer in the region of 4,000~400 cm^{-1} with a resolution of 2 cm^{-1}.

2. Quantification of the Discharge Products

2.1. Preparation of discharged cathodes

The titration quantification conducted here is modified based on previous work.[3] A cathode was removed from the discharged cell and placed in a glass vessel in an Ar-filled glove box without exposure to air. Typically, the time between the end of cell discharge and cathode extraction was less than 0.5 h. The vial was then sealed with a silicone septa lid and transferred out of the glove box. 20 mL of ultrapure water (18.2 MΩ cm, Millipore) was injected into the sealed vial using a syringe. The vial contents were then vigorously shaken for 30 seconds. The resulting solution was used for the quantification of Li_2CO_3, Li_2O_2 and LiOH.

2.2. Li_2CO_3 calibration

To calibrate the amount of Li_2CO_3, lithium carbonate (Li_2CO_3) solutions with known quantities were prepared by dissolving Li_2CO_3 (>99.99%, Macklin) of different mass in ultrapure water. The diluted Li_2CO_3 solution (0.5 ml) was injected into the cell using a syringe under an Ar flow (2 ml/min). When a stable CO_2 partial pressure was obtained after injecting Li_2CO_3 solution, and then HCl (1 M, 1 mL) was injected into the cell to obtain CO_2 signal. The amount of Li_2CO_3 was correlated to the amount of evolved CO_2 (Equation S1) which was detected by the mass spectrometer (Figure S2a). By integrating the amount of evolved CO_2, the total number of moles of Li_2CO_3 was calculated using the standard curve (Figure S2b).

$Li_2CO_3 + 2HCl \rightarrow 2LiCl + H_2O + CO_2$ **Equation S1**

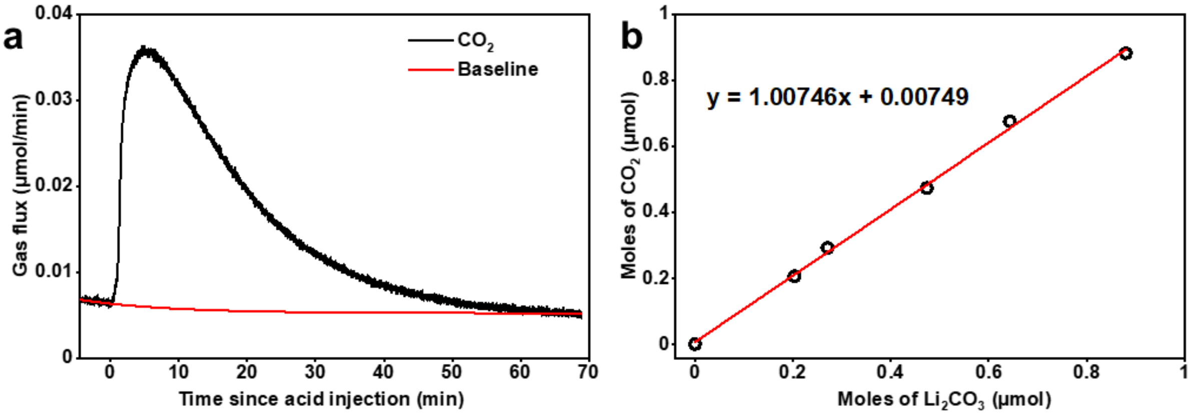

Figure S2. (a) Representative CO_2 evolution after injection of 1 M HCl into a vessel containing diluted Li_2CO_3 solution. (b) The standard curve for Li_2CO_3 quantification.

2.3. Li_2O_2 calibration

The $TiOSO_4$-based ultraviolet–visible titration was used for the quantitative determination of Li_2O_2.[4] Li_2O_2 was firstly hydrolyzed to form LiOH and H_2O_2 (Equation S2), and then $TiOSO_4$ was oxidized with hydrogen peroxide (Equation S3). UV-vis spectrometer (UV-2800, Shanghai Yixin Instrument Co., Ltd.) of the obtained yellow solution (TiO_2SO_4) shown an adsorption peak at 405 nm. To calibrate the amount of Li_2O_2, a series of H_2O_2 solutions were prepared by dissolving 30 wt.% H_2O_2 solution (SCR) of different volume in ultrapure water. To ensure the precision of the diluted H_2O_2 concentration, $KMnO_4$ titration was used to further calibrate. Different concentrations of H_2O_2 (1 mL) and $TiOSO_4$ (1 mL, 15 wt.% in dilute sulfuric acid, Rhawn) were added to quartz cuvette and the corresponding UV-vis spectrum of mixed solution was obtained, as shown in Figure S3a. By measuring the absorbance of peak at 405 nm, the number of moles of Li_2O_2 was calculated using the standard curve (Figure S3b).

$Li_2O_2 + 2H_2O \rightarrow 2LiOH + H_2O_2$ **Equation S2**

$H_2O_2 + TiOSO_4 \rightarrow TiO_2SO_4 + H_2O$ **Equation S3**

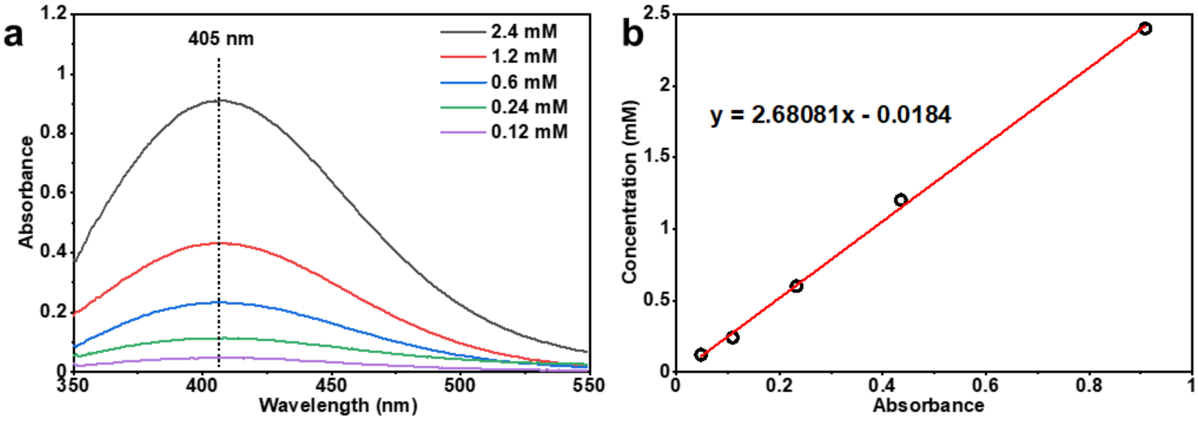

Figure S3. (a) UV-vis spectrum of TiO_2SO_4 complex prepared with different concentrations of H_2O_2. (b) The standard curve for Li_2O_2 quantification.

2.4. LiOH calibration

LiOH was titrated with HCl (Equation S4). HCl solutions with known quantities were prepared by diluting 37 wt.% HCl solution (SCR) in ultrapure water. The precise concentration of HCl was determined by titration using standard Na_2CO_3 solution (0.05 M, Knuox). Different known concentrations of lithium hydroxide (LiOH) solution were prepared by diluting LiOH (>99.9%, Macklin) of different mass and dissolving in ultrapure water. The titration end point was determined by the color change (from blue to pink, as shown in Figure S4a) of a double indicator (Bromocresol Green/Methyl Red, Collins). The standard curve (Figure S4b) was calculated by adding the number of moles of HCl to react with LiOH of known quantities to reach the color change (titration end point). The presence of Li_2CO_3 and Li_2O_2 would consumed HCl solution, while a prior knowledge of the total amount of Li_2CO_3 and Li_2O_2 in solution enables us to calculate the total amount of LiOH. As shown in equation S5, the hydrolysis of three components (n(Li_2CO_3), n(Li_2O_2) and n(LiOH)) would determine the total alkalinity (n(all)) of the solution.

$LiOH + HCl \rightarrow LiCl + H_2O$ **Equation S4**

$n(LiOH) = n(all) - 2n(Li_2CO_3) - 2n(Li_2O_2)$ **Equation S5**

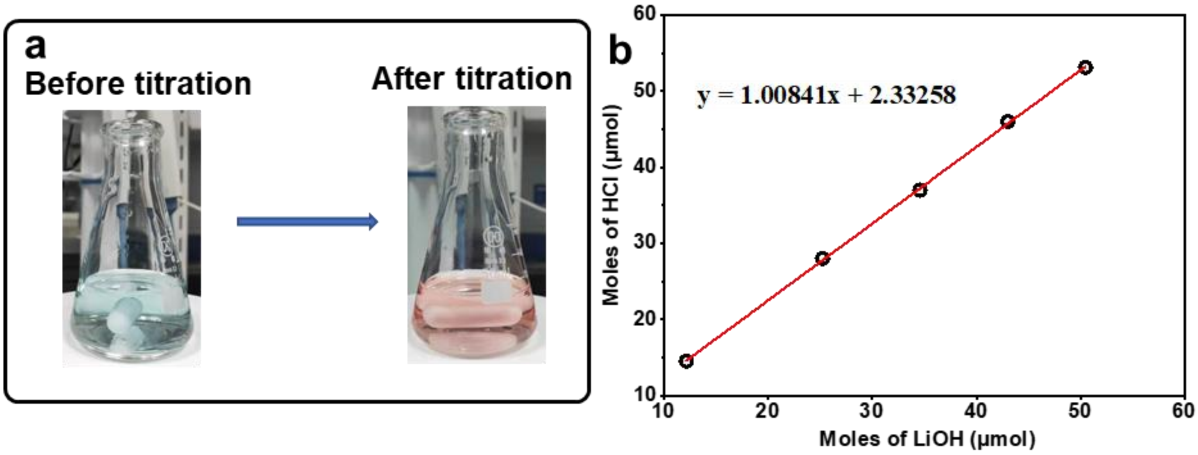

Figure S4. (a) Color change during alkaline titration. (b) The standard curve for LiOH quantification.

2.5. The original processed data of quantified discharge products

Table S1. Quantification of Li_2CO_3, Li_2O_2 and LiOH in discharge products via titration.

System	Conditions	Li_2CO_3 [μmol]	Li_2O_2 [μmol]	LiOH[a] [μmol]
Li_2O_2-based batteries	1th-pure O_2	1.7	15.9	1.6
	1th-dry air	2.8	14.2	2.0
	4th-dry air	3.6	11.7	1.4
	7th-dry air	5.1	5.5	1.7
LiOH-based batteries	1th-pure O_2	0.1	[b]	17.8
	1th-dry air	0.4	[b]	19.2
	4th-dry air	1.8	[b]	15.7
	7th-dry air	2.2	[b]	15.8
	1th-ambient air	0.4	[b]	16.9
	4th-ambient air	1.8	[b]	16.3
	7th-ambient air	2.1	[b]	15.7

[a] In order to obtain the percentage of different discharge products in total discharge products (only include Li_2CO_3, Li_2O_2 and LiOH), all values of LiOH are divided by 2; [b] For LiOH-based batteries, the concentration of Li_2O_2 is too low to be detected via UV-vis spectrum.

2.6. Evaluation of measurement errors in titration

Table S2. The result of measurement error.

Serial number	Weighing value			Measured value			Measured error		
	Li_2O_2/mg	Li_2CO_3/mg	LiOH/mg	Li_2O_2/mg	Li_2CO_3/mg	LiOH/mg	Li_2O_2/%	Li_2CO_3/%	LiOH/%
1	1.4	0.6	0.8	1.21	0.63	0.87	13.57	5	8.75
2	1.3	0.8	1.6	1.19	0.87	1.77	8.46	8.75	10.63
3	1.3	8.7	2.7	1.27	8.73	3.14	2.31	0.34	16.30

In order to further confirm the accuracy of our titration methods, mixtures of Li_2O_2, LiOH and Li_2CO_3 with different known masses had been weighed, and subjected to titration to evaluate the accuracy. The results are given in **Table S2**: the errors for Li_2O_2, Li_2CO_3, LiOH are <10%, <15%, and ~15%, respectively. Our measurement error is similar to those in others' report.[5]

3. Characterization of Ru/SP

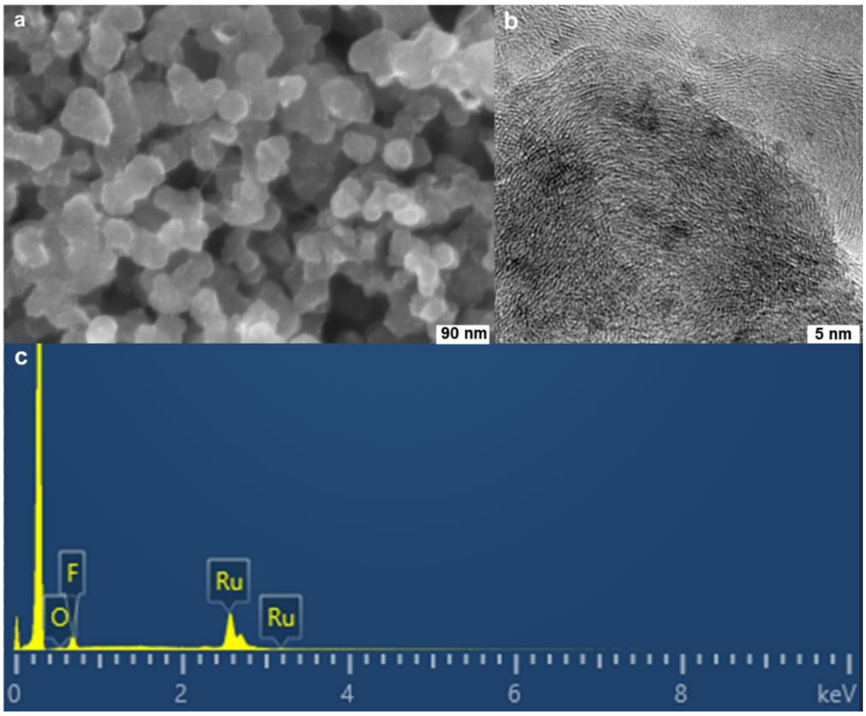

Figure S5. SEM (a), TEM (b) of the synthesized Ru/SP and EDX spectrum (c) of the pristine Ru/SP/PTFE electrode.

SEM images of the synthesized Ru/SP powder (Figure S5a) showed that Ru particles were homogeneously dispersed on the carbon, and TEM images (Figure S5b) of synthesized Ru/SP powder revealed that particulate Ru catalyst were of less than 5 nm. EDX spectra (Figure S5c) of pristine Ru/SP/PTFE electrodes confirmed the peaks of Ru element at 2.6 and 3.2 keV. These results demonstrate that a homogeneous dispersal of Ru catalyst particles (5 nm) in pristine electrodes.

4. Li-dry air battery using Ru/SP electrodes with anhydrous electrolytes

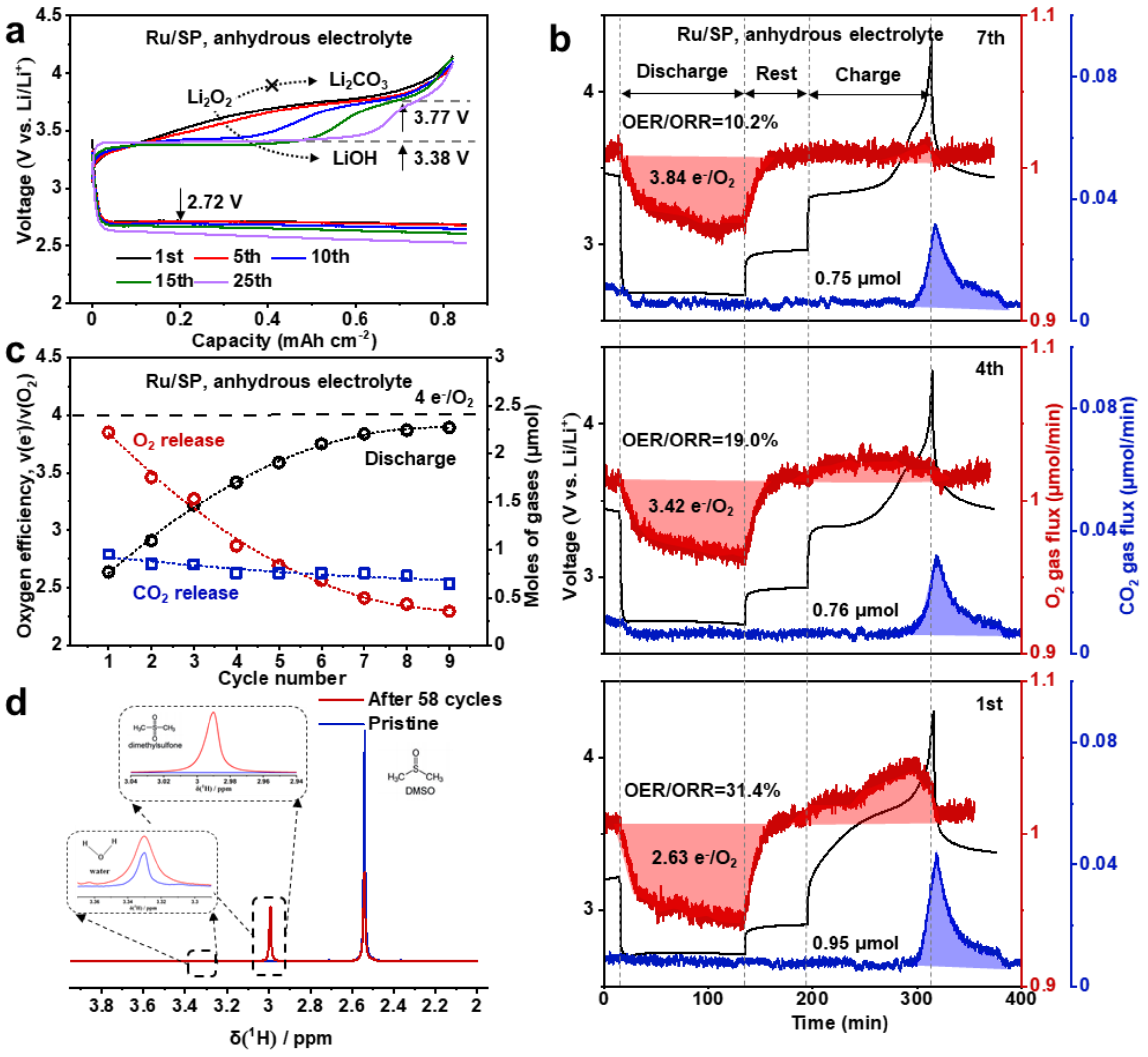

Figure S6. Investigating the electrochemistry of lithium-air cells using Ru/SP cathodes and an anhydrous 1 M LiTFSI/DMSO electrolyte under dry air. This cell was constructed based on a strictly sealed design, to prevent water in ambient air from entering the system. (a) Electrochemical profiles of the cell over extended cycles. (b) Voltage profiles and the corresponding O_2 (m/z=32), CO_2 (m/z=44) signals during cycling of the cell in dry air. (c) Analysis of OEMS data corresponding to the cell electrochemistry in (a) illustrating the trends for O_2 and CO_2 consumption and evolution with cycle number. (d) ^{1}H NMR analysis of the cycled and pristine electrolytes.

A Li-dry air battery was constructed using Ru/SP electrodes with anhydrous 1 M LiTFSI/DMSO to investigate the electrochemistry in a strictly sealed chamber. As shown in Figure S6a, the electrochemical profiles of the cell, especially the charging process, exhibited a gradual change over cycling. The voltage gradually decreased, forming a lower charge plateau at ~3.38 V, which became dominant after 25 cycles. Eventually, the charge profile resembles that of a cell using a wet electrolyte (Figure 2d). More information could be obtained from the corresponding OEMS characterization. The electrons per reduced O_2 and OER/ORR of the cell have changed from 2.63 e^-/O_2 and 31.4% at 1st cycle to 3.84 e^-/O_2 and 10.2% at 7th cycle respectively (Figure S6b). Quantitative analysis of OEMS data (Figure S6c) in first 9th cycle showed that as the electron/O_2 molar ratio increased from 2 to 4, O_2 and CO_2 evolution were both reduced, consistent with LiOH formation and decomposition becoming the dominant reactions during cycling. Because this cell was in a strictly sealed system, any rise in water in the cycled electrolyte should be originated from the battery chemistry itself. Previous studies [6-7] have suggested that the formation of LiOH in anhydrous DMSO electrolyte was due to the nucleophilic attack of $O_2{\cdot}^-$ to the electrolyte, and subsequent LiOH decomposition on charging would generate water due to Ru catalysts.[1] ^{1}H NMR analysis (Figure S6d) of the cycled electrolytes further confirmed that indeed the water signal has increased compared to that of a pristine electrolyte. It therefore suggests that even in an anhydrous cell using Ru/SP catalysts, the cell reaction initially being Li_2O_2-dominated will be converted to LiOH in nature.

5. Characterization of discharged products via ^{7}Li solid state NMR and FTIR

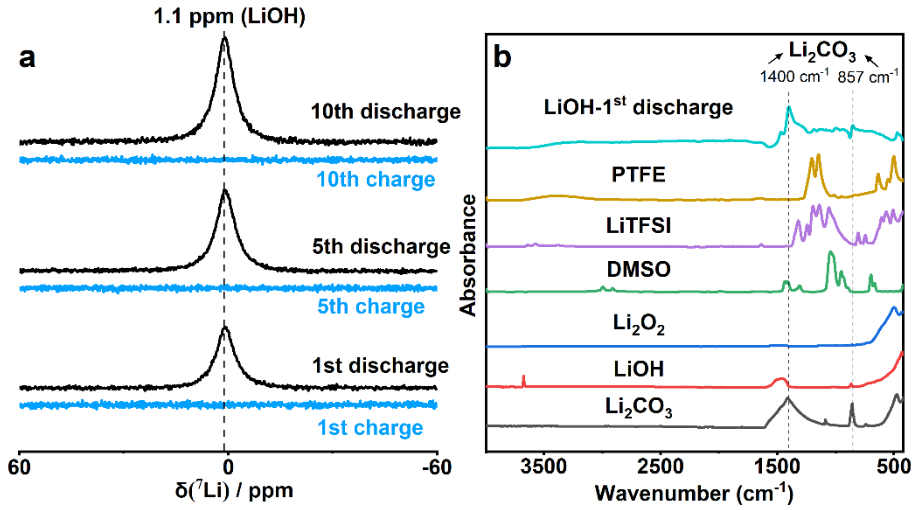

Figure S7. (a) Magic angle spinning ^{7}Li solid state NMR spectra and (b) FTIR spectra of LiOH-based cathodes at discharged/charged states of different cycle numbers. The discharge capacity of all batteries is 1 mAh. A single resonance at 1.1 ppm in the ^{7}Li spectrum suggests LiOH is the dominant discharge product. The peaks at 857 and 1400 cm^{-1} of FTIR represent the formation of Li_2CO_3.

In order to further characterize the discharged products, ^{7}Li solid state nuclear magnetic resonance (NMR) and Fourier transformed infrared spectroscopy (FTIR) measurements have been performed to characterize the formation and decomposition of discharge products. In Figure S7a, 6 cells terminated at discharged/charged states of different cycle numbers were prepared and subjected them to ^{7}Li solid state NMR tests. Consistent experimental parameters (number of scans, discharge/charge capacities) and sufficient recycle delay were applied to allow quantification of the cell reaction. A single resonance at 1.1 ppm (Figure S7b) for ^{7}Li, characteristic of LiOH, was observed in discharged samples and completely disappeared after recharge. This additional result further supports that the cell reaction is predominantly based on LiOH formation and decomposition, consistent with the rest of our report. The 1st discharged

cathode was prepared to characterize the discharged products via FTIR. Although the peaks of Li_2CO_3 at 857 and 1400 cm^{-1} can be observed (Figure S7b), FTIR characterization of discharged cathode suffers from difficulties for quantification (only sampling a small fraction of the cathode). Furthermore, the most intense peaks situate at the far infrared region (200-600 cm^{-1}) for all potential products (LiOH, Li_2CO_3, Li_2O_2) and are difficult to be discerned and differentiated in common mid-IR spectra. Therefore, in this work we primarily rely on quantitively analysis such as chemical titration, OEMS and the newly added solid state NMR to verify the nature of the cell reaction.

6. CO_2 accumulation impact on Li_2O_2-based electrochemistry

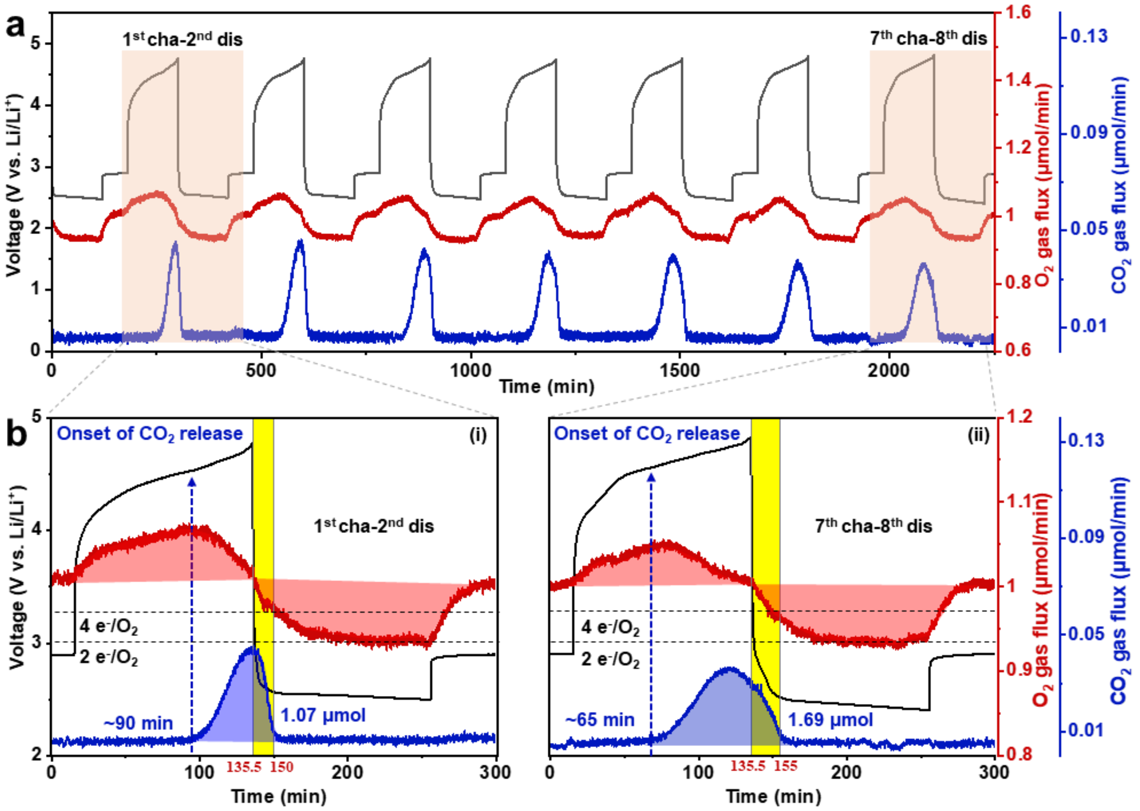

Figure S8. Gases consumption and evolution during cell cycling in air using OEMS. (a) Discharge and charge profiles and the corresponding O_2 (m/z=32), CO_2 (m/z=44) signals of a cell using a SP cathode and 1 M LiTFSI/DMSO under dry air. (b) Enlarged view of the 1st charge-2nd discharge (i) and 7th charge-8th discharge regions (ii) of OEMS. The electron number per reduced O_2 gas molecule are marked with the black dashed lines. The blue dashed line denotes the onset of CO_2 release. Regions shaded by yellow color (panel i: 135.5-150 min, ii: 135.5-155 min) represent the time periods for electrochemical reduction of CO_2.

To reveal the impact of CO_2 accumulation on Li_2O_2-based electrochemistry over extended cycles, the corresponding O_2 (m/z=32), CO_2 (m/z=44) signals during cycling were obtained via OEMS. As shown in Figure S8a, there was no resting periods between charge and the following discharge, which was attempted to be kept consistent with Figure 2c. In order to focus on the

details, regions representing the transition from 1st charge to 2nd discharge (Figure S8b (i)), and from 7th charge to 8th discharge (Figure S8b (ii)), have been enlarged.

As shown in Figure S8b(i), O_2 evolution occurred at the beginning of the 1st charge, but started to decrease beyond 2/3 of the charge capacity (indicated by the dashed arrow), at which point CO_2 evolution began to rise. During the following discharge, oxygen consumption and a concomitant CO_2 signal drop were observed. Of note, a small first step in the O_2 consumption profile was recorded, which corresponded to a level of four electrons per reduced O_2; moreover, a rapid drop in the CO_2 signal (bending of the CO_2 profile, as more clearly revealed in Figure S8b(ii)) was observed in accordance to a small discharge step in the electrochemical profile. These observations suggest that in the presence of higher CO_2 concentrations, cell discharge was initiated by electrochemical CO_2 reduction, probably via $CO_2 + O_2 + 4Li^+ + 4e^- \rightarrow 2Li_2CO_3$.[8-12]

During the decomposition of Li_2CO_3 on charging, the O_2 released was far below the value expected by the reaction stoichiometry above, the O_2 signal falling to zero when CO_2 evolution peaked, suggesting that L_2CO_3 formation is not reversible[9,11,13-14]. At the 7th charge, the amount of O_2 evolution decreased, whereas Li_2CO_3 decomposition and CO_2 evolution increased considerably, implying accumulation of Li_2CO_3 upon cycling. It is worth noting that the length of the discharge plateau related to electrochemical CO_2 reduction in OEMS experiments was much shorter than that in Figure 2c, which is likely due to the constant working gas flow through the cell rapidly dissipating the released CO_2. In the absence of a dynamic gas flow (Figure 2c), the CO_2 concentration localized at the electrode-electrolyte interface would be much higher, promoting direct CO_2 reduction for a larger portion of the discharge process. Therefore, the main route for Li_2CO_3 in a cell with SP cathodes and anhydrous electrolytes (Li_2O_2-based electrochemistry) in air is primarily via direct electrochemical reduction of CO_2.

7. The impact of resting time on CO_2 evolution during recharging

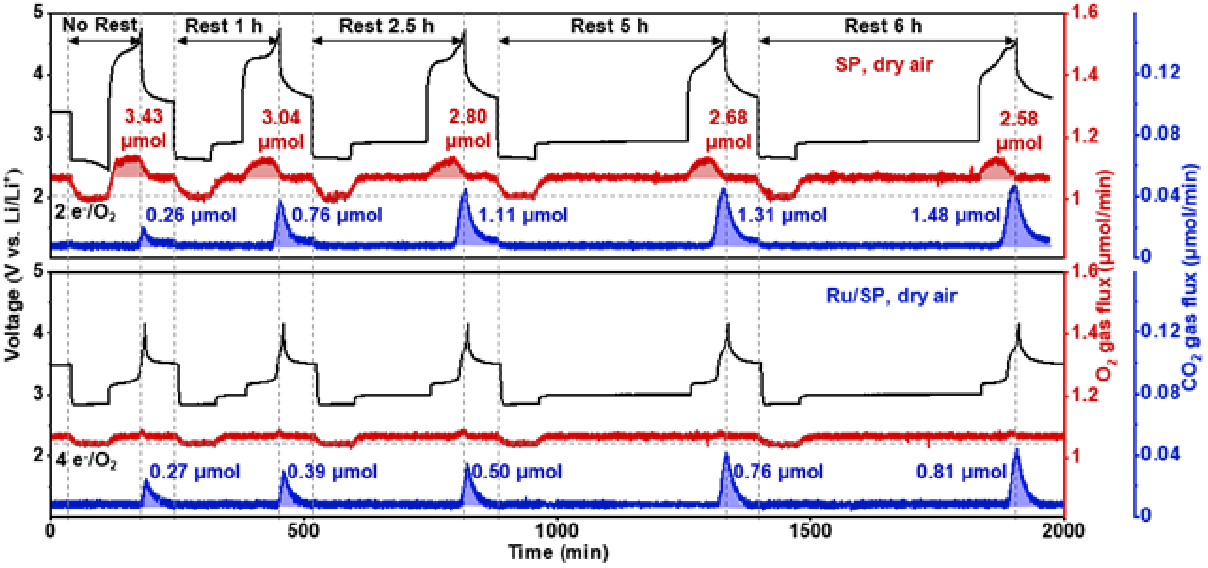

Figure S9. Evaluating the impact of resting time on CO_2 evolution on recharging via OEMS. Discharge and charge profiles and the corresponding O_2 (m/z=32), CO_2 (m/z=44) signals of cells using different cathodes and electrolytes under dry air: (i) SP cathode with 1 M LiTFSI/DMSO and (ii) Ru/SP cathode with 1 M LiTFSI/DMSO with 5 vol% H_2O. Levels for two electrons per reduced O_2 (i) and four electrons per reduced O_2 (ii) are marked using black dashed lines.

In order to compare the chemical stability of the two discharge products (Li_2O_2 and LiOH) under dry air, the resting period between battery discharges and charges has been varied from 0 to 6 hours. The corresponding O_2 (m/z=32), CO_2 (m/z=44) signals during cycling was obtained via OEMS. As shown in Figure S8i, for cells using SP cathodes and anhydrous DMSO electrolytes, the discharge process showed around two electrons per reduced O_2, consistent with dominant Li_2O_2 formation. With the increase of rest time (from 0 to 6 h), O_2 release was decreased (from 3.43 to 2.58 µmol), and CO_2 release was increased (from 0.26 to 1.48 µmol). For cells using Ru/SP cathodes and DMSO electrolytes with 5 vol% H_2O (Figure S9ii), however,

the discharge process showed around four electrons per reduced O_2, consistent with dominant LiOH formation. No O_2 release was observed on subsequent charge processes due to $DMSO_2$ formation, consistent with previous reports.[1] Meanwhile, with the increase of resting time, CO_2 release was also increased (from 0.27 to 0.81 μmol) for Ru/SP case. Nonetheless, for the same resting period, the cell with SP cathode revealed more CO_2 evolution than that with Ru/SP cathode, which is consistent with the higher thermodynamic driving for CO_2 reaction with Li_2O_2 than LiOH to form Li_2CO_3.[15]

8. Factors affecting the fraction of Li_2CO_3 formation

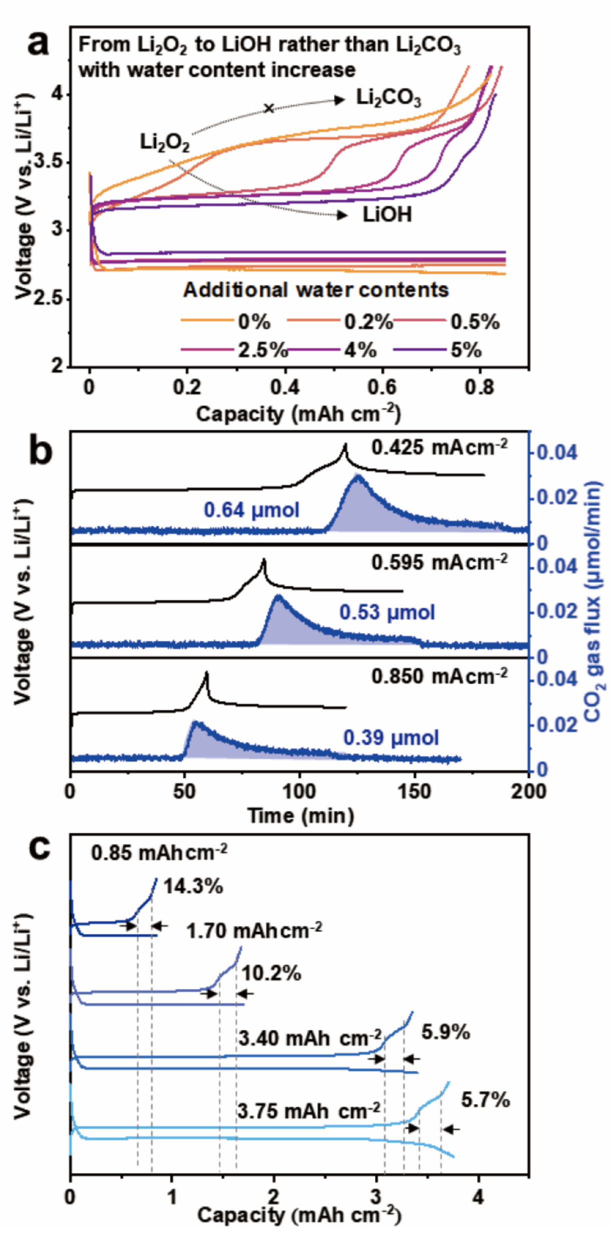

Figure S10. (a) The discharge-charge curves (the 1st cycle) of Li-ambient air cells as a function of water contents in 1 M LiTFSI/DMSO electrolyte; Ru/SP cathodes were used at 0.425 mA/cm^{-2}. (b) The effect of cycling rates on the amount of CO_2 evolution during charge, where the capacities are kept the same for all three cases. (c) Effects of depth of discharge on the electrochemical fraction related to Li_2CO_3 decomposition during recharging.

9. XRD characterization for Li metal exposed in ambient air

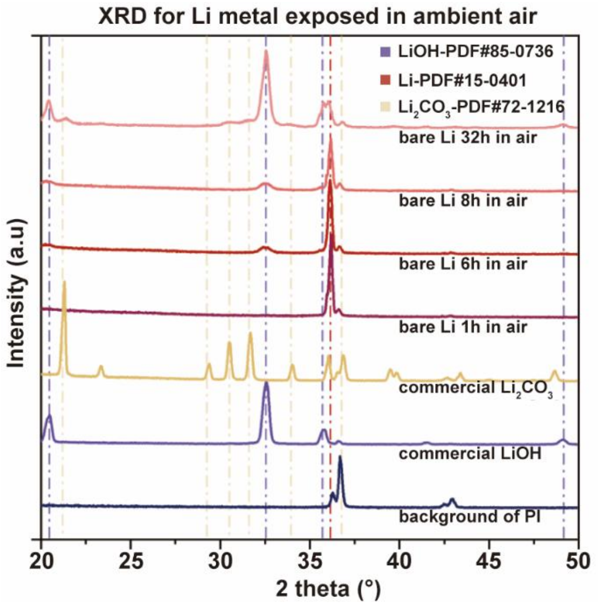

Figure S11. Evolution of XRD patterns acquired from a lithium disc exposed in air for different durations in ambient air. The XRD patterns of the sample holder and relevant reference compounds are also presented for comparison.

For a fresh lithium metal, the diffraction pattern featured two dominant Bragg reflections at 36.8 and 52.0 degrees. After 6 hours, new peaks associated with LiOH could be seen, which continued to intensify and had become the main feature in the diffraction pattern after a day of exposure. At this point, weak reflections linked to Li_2CO_3 formation could also be identified. These observations imply that moisture in air is the most aggressive component that react with lithium metal, forming LiOH as the prevailing surface product.

10. Compartmental Li-air full cell using Ohara ceramic glass

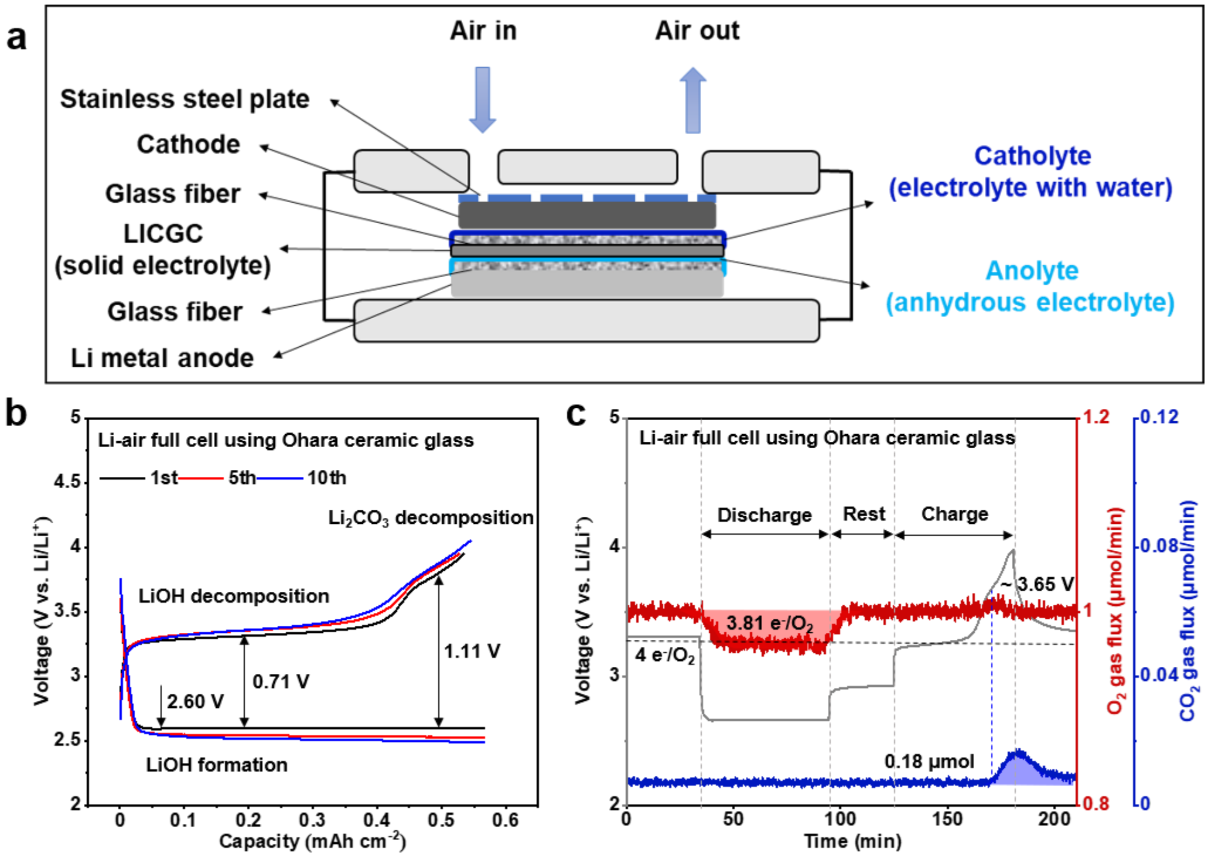

Figure S12. (a) A schematic illustration of Li-dry air Swagelok-type full cell using an Ohara ceramic glass to separate the anode and cathode compartments. (b) Voltage profiles of this full cell at 0.34 mA cm^{-2}. (c) Voltage profiles and the corresponding O_2 (m/z=32), CO_2 (m/z=44) signals of this full cell during operation.

We initially employed a dual compartmental design by an Ohara ceramic glass separating the anolyte and catholyte parts (Figure S12a). In dry air, this setup permitted stable electrochemical cycles (Figure S12b), the discharge-charge profile resembling that of half cells (Figure 2d). OEMS measurement further supported that the cell reaction was based on an ORR, consistent with LiOH formation. Nevertheless, this dual compartmental setup is not adequate to enable a stable cycling in real air. The battery failed in just two days, because of lithium corrosion by air.

11. Impendence for LATP discs and LATP-wax composite

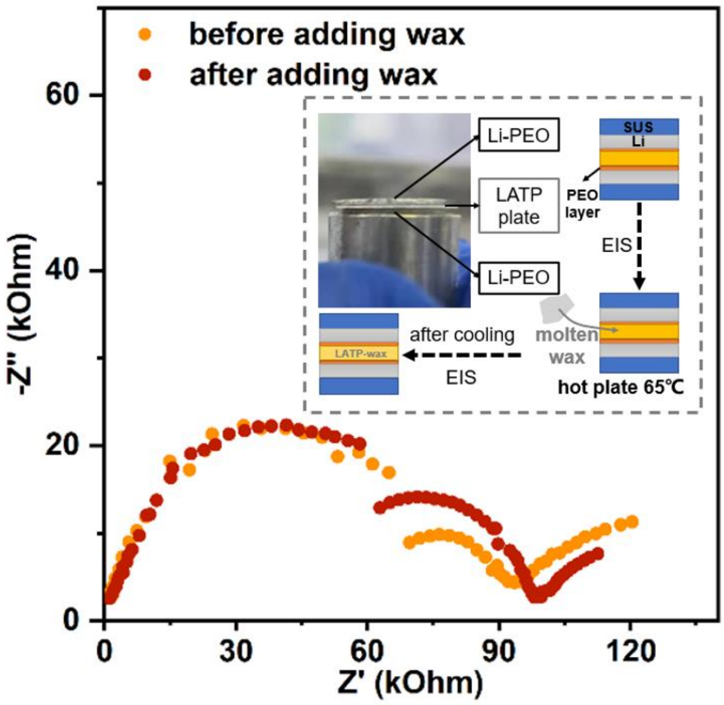

Figure S13. Electrochemical impedance spectra of Li-PEO-LATP-PEO-Li before (orange) and after (red) filling the wax at 40 ℃. The testing methods scheme are inserted in the upper right corner of the picture.

To investigate whether wax increases the resistance to Li^+ diffusion, we compared the EIS results obtained on two setups, one with PEO-LATP and the other with PEO-LATP-wax electrolyte (*note that the PEO-LATP-wax was prepared from the same PEO-LATP disc*) in an all-solid-state Li symmetric battery (see scheme in Figure S13). The electrolyte resistance in both cases were found to be very close (slight variation may be due to different pressures applied to the battery during measurements), indicating that Li^+ diffuse across the protective layer via LATP-PEO and wax does not induce additional impedance (Figure S13). For ease of practice and improved robustness of the setup, a thicker solid-state protective layer was applied here, and hence the unusually higher resistances observed.

12. Protection strategy of Li anodes

7a37d268446ccb8808891a5f5de1472a.mp4

Movie S1. Videos comparing the air-stability and reactivities to wet electrolytes/water of Li anodes. Vigorous gas bubbling has been observed in all cases except for the lithium anodes protected by the PEO-LATP-wax composite layer.

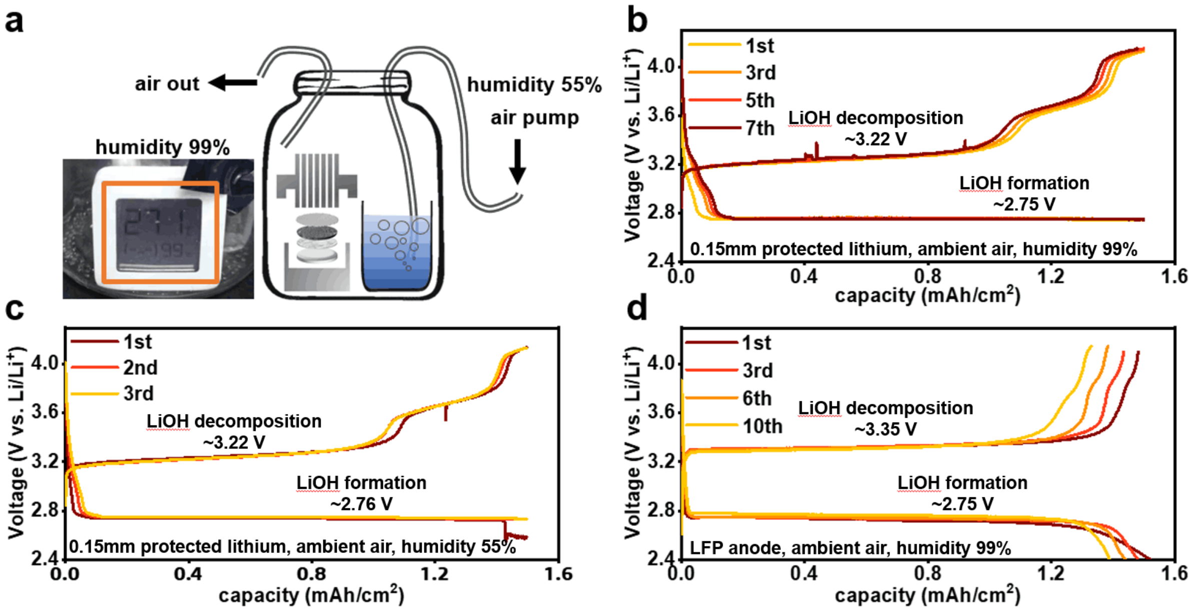

Figure S14. Electrochemical performance of lithium-air cells at 99% humidity. A device that maintains 99% humidity made by ourselves consisting of air pump and hygrometer (a). Voltage profiles using protected 0.15 mm protected lithium (b) or LFP as counter electrode (d) and 1 M LiTFSI/DMSO with 5 vol% H_2O in 99% humidity air. Voltage profiles of cells using 0.15 mm protected lithium and 1 M LiTFSI/DMSO with 5 vol% H_2O in 99% humidity air (c).

In order to study the potential of high humidity operation, we constructed a lithium-air full cell using a thin lithium foil (0.15 mm), a wet (5 vol%) LiTFSI/DMSO electrolyte and Ru/SP cathode, cycling in 99% maximum humidified air (Figure S14a**)**. When the humidity was

increased to 99%, the voltage profiles showed no difference (Figure S14b) from the same cell operating in ambient air (Figure S14c). A fully discharged/charged cell to a larger areal capacity is also presented in Figure S14d These additional experimental results confirmed the validity of the lithium protection strategy by PEO-LATP-wax, and further verified the robustness of the LiOH formation/decomposition mechanism against air and water.

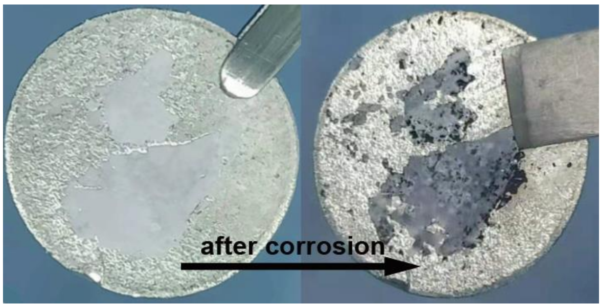

Figure S15. Optical images showing the color change of a LATP layer after it has been in direct contact with a lithium metal in an Ar glove box.

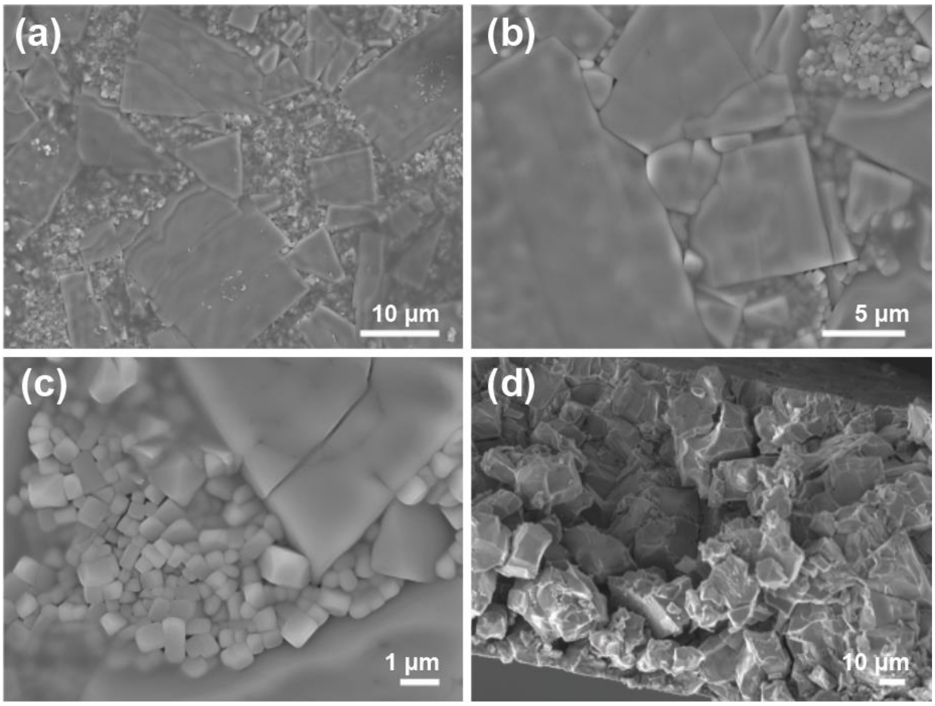

Figure S16. SEM images illustrating the top surfaces (a-c) and cross-section (d) of the protective layer, retrieved from a lithium-air cell cycled in ambient air until the cell failed.

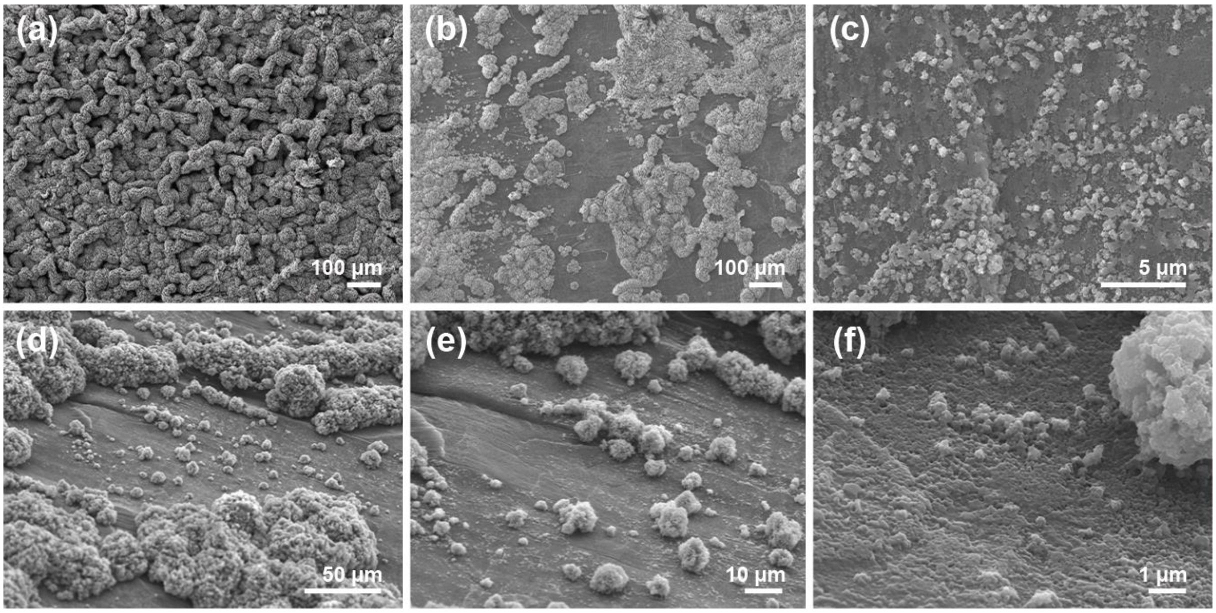

Figure S17. SEM images of the PEO-wax (25:100 in weight) protected Li anode from a cell that has been cycled in air until cell failure.

Although a PEO-wax protective layer could render the lithium anode chemically stable in air and water, as previously reported, we found it could not sustain stable electrochemical cycles of a lithium-air battery in neither wet electrolytes nor in ambient air. Extensive nucleation and growth of lithium dendrite have been observed (Figure S17a-c) on the PEO-wax layer after cycling. In some regions, clear evidence of dendrites penetrating through this protective was found (Figure S14d-f). The electrochemical profile of a full cell using LiTFSI/DMSO with 5% H_2O gradually reverted to that with an anhydrous electrolyte (Figure S17). The corresponding OEMS results further suggested that the reaction had gradually transformed from LiOH formation to Li_2O_2 formation, as evidenced by the increasingly larger O_2 consumption during discharge and more CO_2 and O_2 evolution on recharging.

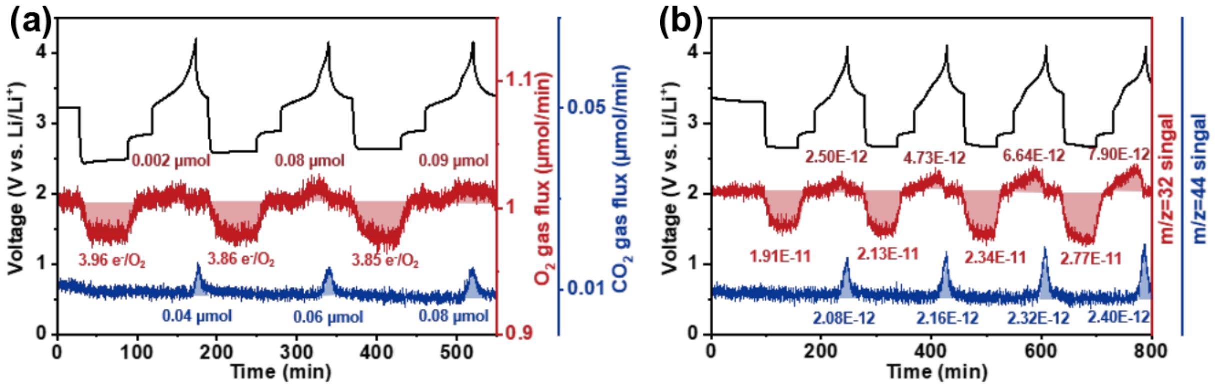

Figure S18 OEMS measurements of a lithium-air battery using PEO-wax (25:100) protected lithium anode, Ru/SP cathode and wet 1 M LiTFSI/DMSO electrolytes (5 vol% H_2O), where the gas consumption and evolution signals have been quantified and calibrated for the 1st-3rd cycles (a). In the following four cycles (b), peak areas have been integrated (but not calibrated) to show the trend in gas intake and release during cycling.

13. Characterization for cycled electrolyte and air cathode

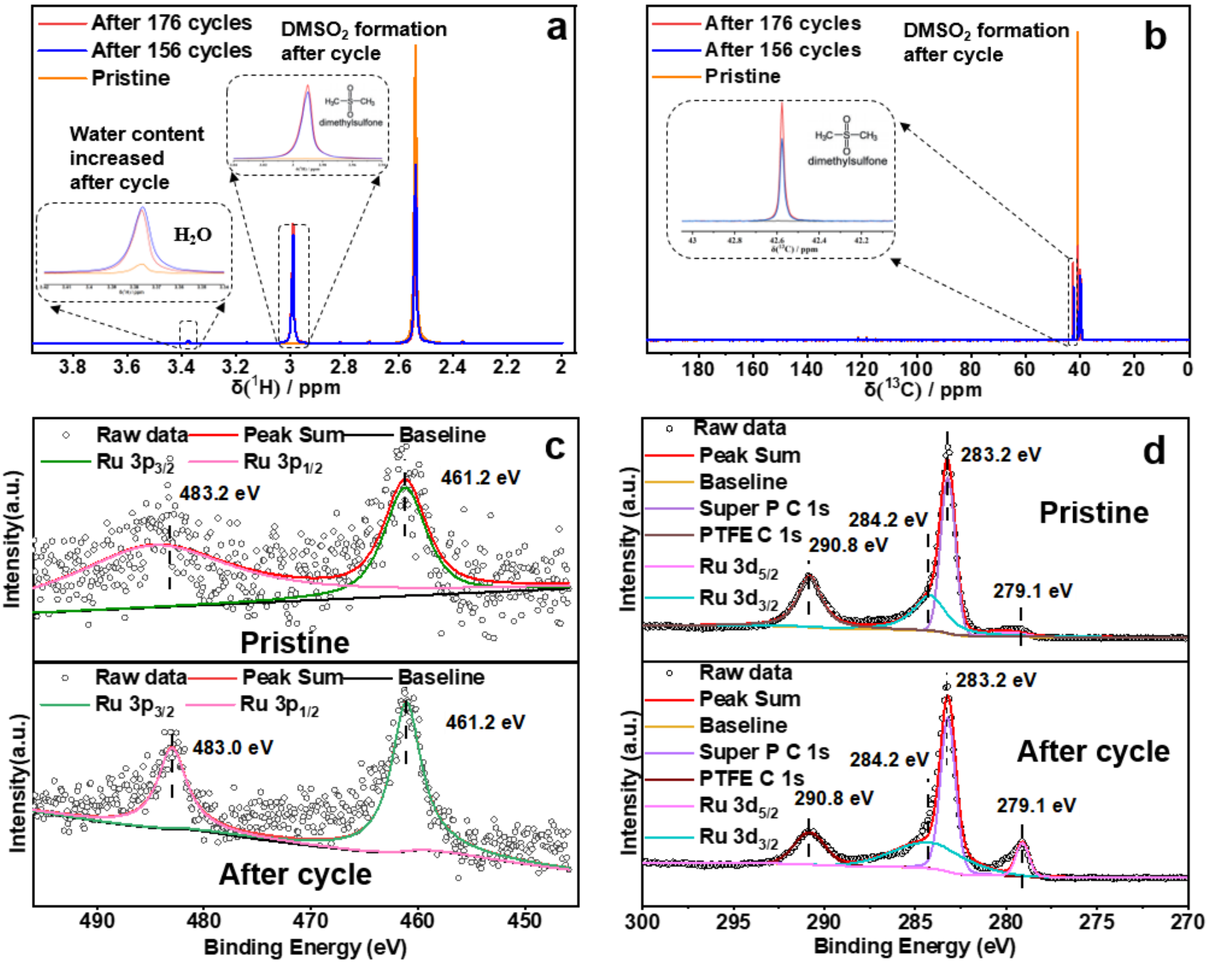

Figure S19. ^{1}H NMR (a) and ^{13}C NMR (b) measurements of the residual electrolyte from cells (Ru/SP, 1 M LiTFSI/DMSO and 5 vol% H_2O) that have been subjected to extended cycles. Ru 3p (c) and C 1s (d) XPS spectra of the cycled (obtained from the same cycled cells as for a and b) and pristine (top) electrodes.

Previous work[1] by some of the authors showed by using ^{1}H, ^{13}C and ^{17}O solution NMR that the oxygen in $Li^{17}OH$ (isotopically labelled) ends up as $DMS^{17}O_2$ in the electrolyte, i.e., DMSO was oxidized to $DMSO_2$ during LiOH decomposition. To clarify the decisive factor limiting the cycle life of the full lithium-air battery in this work, the electrolyte (separator) and the Ru/SP cathode from a cell cycled after 156 and 176 cycles have been investigated using solution NMR

and XPS, respectively. ^{1}H and ^{13}C NMR measurements of a dried-out separator showed that after cycling, considerable $DMSO_2$ formed (resonances at 2.99 ppm and 42.6 ppm for $\delta(^{1}H)$ and $\delta(^{13}C)$, respectively),[1] which was absent in the pristine electrolyte. This indicates that DMSO solvent in the electrolyte appeared to be oxidized during repeated charging (as there was no oxygen evolution). On the contrary, the Ru/SP cathode after cycling revealed little chemical change, as evidenced by XPS results; this reiterates a better interfacial stability between LiOH and carbon cathodes. The surface of the mesoporous cathode was not covered by a large amount of solid side reaction products (Figure 7f(iii-vi)), unlike the typical case cycling via Li_2O_2 formation. In addition, ^{1}H measurement shows that the water content in the electrolyte increased after cycling (resonance at 3.38 ppm for $\delta(^{1}H)$), which may result from the charging reaction continually regenerating water[1] and some ingression of moisture from the ambient air over time. Therefore, the failure of the full cell was likely related to electrolytes being depleted rather than cathode clogging or H_2O being depleted.

14. Tables comparing our work with others in literature

Table S3 shows that hardly any prior studies have demonstrated a highly rechargeable lithium-air full cell could operate under the harsh conditions investigated in this work. In terms of electrochemical performance (cycle life, areal/specific capacity), the results shown in this study are competitive to some of the best work reported (Table S4).

Table S3. A summary of testing conditions in literature to evaluate the tolerance of protected lithium

**Report**	**Journal**	**Protective Layer**	**Battery System**	**Working Conditions of Air Battery**				**Ref.**
				**Electrolyte**	**Water**	**Humidity**	**Atmosphere**	
**1**	2020 Adv. Mater.	hydrophobic graphene	LFP and NCM-811					[16]
**2**	2017	PDMS film	LFP					[17]

	Adv. Mater.							
**3**	2021 Adv. Mater.	PDDA-TFSI	LFP and NCM-811					[18]
**4**	2018 Adv. Mater.	$GeCl_4$ pretreatment	$Li\text{-}O_2$ (Li_2O_2)	1 M LiTFSI TEGDME and 0.5 M LiTFSI IL	0.1-1%	45%	O_2	[19]
**5**	2018 Adv. Mater.	boric acid	$Li\text{-}O_2$ (Li_2O_2)	1 M LiTFSI DMSO	0	0	O_2	[20]
**6**	2020 Sci. Bull.	cross-linked PVDF–HFP	half cell					[21]
**7**	2019 Sci. Bull.	WAX-PEO	Li-S					[22]
**8**	2019 Nat. Commun	graphite fluoride	LFP					[23]
**9**	2019 Energy Storage Mater.	tetraethyl orthosilicate	$Li\text{-}O_2$ (Li_2O_2)	1 M $LiCF_3SO_3$ TEGDME	0	0	O_2	[24]
**10**	2020 Energy Storage Mater.	silane mixed solution	Li-Air (Li_2O_2)	0.8 M LiTFSI and 0.5 M $LiNO_3$ in PYR_{14}TFSI/DMSO (2:8, in vol)		15%	air	[25]
**11**	2020 Angew. Chem. Int. Ed.	PEO-Upy	LFP and NCM					[26]
**12**	2019 Angew. Chem. Int. Ed.	TPU-SiO_2	Li-Air (Li_2O_2)	1 M $LiCF_3SO_3$ TEGDME	0		air	[27]
**13**	2019 Adv. Funct. Mater.	triethylaluminum (TEAL)	$Li\text{-}O_2$ (Li_2O_2)	1 M LiTFSI TEGDME and 0.5 M LiTFSI IL	0		O_2	[28]
**14**	2017 Adv. Energy Mater.	polyisobutylene (PIB), SiO_2	$Li\text{-}O_2$ (Li_2O_2)	0.5 M LiTFSI 1-methyl-3propylimidazolium bis(trifluoromethylsulfonyl) imide	0		O_2	[29]
**15**	2021 Adv. Sci.	CVD graphene	Li-Air (Li_2O_2)	1 M LiTFSI TEGDME	0		air	[30]
**16**	2020 ACS Energy Lett.	SiO_2-PVDF	NCM-523					[31]
**17**	our work	PEO-LATP-WAX	Li-Air (LiOH)	1 M LiTFSI DMSO	5%	55-99%	ambient air	

Table S4. A summary of performances of lithium air batteries using solid-state Li^+ conductive electrolyte under ambient air in the literature.

Report	Year	Battery Structure	Specific Current	Areal Current	Specific Capacity	Areal Capacity	Cycles	Ref.
1	2012 Electrochem. Commun.	Pt | 0.1 M H_3PO_4 + 1 M LiH_2PO_4 | LTAP | 1 M $LiPF_6$ in EC/DEC | Li	Not mentioned	0.5 mA cm^{-2}	221 mAh g^{-1}	Not mentioned	20	[32]
2	2013 Carbon	CNT sponge wetted with IL | LiSICON | IL | Li	100 mA g^{-1}	0.1 mA cm^{-2}	1000 mAh g^{-1}	1 mAh cm^{-2}	16	[33]
3	2013 Nat. Commun.	Gel | LATP | EC-based anolyte | Li	200 mA g^{-1}	0.16 mA cm^{-2}	2000 mAh g^{-1}	1.6 mAh cm^{-2}	30	[34]
4	2014 ACS Appl. Mater. Interfaces	SP+LATP | LATP | Separator immersed with TEGDME | Li	100 mA g^{-1}	0.067 mA cm^{-2}	500 mAh g^{-1}	0.335 mAh cm^{-2}	10	[35]
5	2015 Energy Environ. Sci.	Carbon-coated LATP with silicone-oil films | LATP | Separator immersed with electrolyte (EC/DMC=1:3) | Li	166.67 mA g^{-1}	0.3 mA cm^{-2}	5000 mAh g^{-1}	9 mAh cm^{-2}	50	[36]
6	2015 ACS Appl. Mater. Interfaces	CNT+LAGP | LAGP | Li	400 mA g^{-1}	Not mentioned	1000 mAh g^{-1}	Not mentioned	10	[37]
7	2016 Inorg. Chem.	LiIHPN-LiI@3D-G | LiIHPN-LiI | Li	67 mA g^{-1}	Not mentioned	2740 mAh g^{-1}	Not mentioned	10	[38]
8	2017 J. Power Sources	Pt+CNT | LiOH catholyte | LAGP | $LiPF_6$ in EC-DMC | Li	Not mentioned	0.03 mA cm^{-2}	33 mAh g^{-1}	Not mentioned	140	[39]
9	2018 J. Mater. Chem. A	CNT+RuO_2+LAGP | LAGP | Li	400 mA g^{-1}	0.208 mA cm^{-2}	1000 mAh g^{-1}	0.52 mAh cm^{-2}	27	[40]
10	2018 Adv. Energy Mater.	CNT+RuO_2+LAGP+IL | LAGP | Li	200 mA g^{-1}	Not mentioned	1000 mAh g^{-1}	Not mentioned	30	[41]
11	2018 J. Power Sources	CNTs+LAGP+RuO_2 | LAGP | Li	400 mA g^{-1}	Not mentioned	1000 mAh g^{-1}	Not mentioned	10	[42]
12	2020 J. Power Sources	Ketjen black+Ru |LLZO-based polymer | Li	50 mA g^{-1}	0.05 mA cm^{-2}	300 mAh g^{-1}	0.3 mAh cm^{-2}	50	[43]
13	2021 Nature	CNT | LiXZM | Li	500 mA g^{-1}	0.025 mA cm^{-2}	1000 mAh g^{-1}	0.05 mAh cm^{-2}	150	[44]
14	Our work	SP+Ru |1 M LiTFSI/DMSO with 5 vol% H_2O | wax+LATP+PEO |Li	250 mA g^{-1}	0.25 mA cm^{-2}	500 mAh g^{-1}	0.5 mAh cm^{-2}	176	

15. Reference

[1] T. Liu, Z. Liu, G. Kim, J. T. Frith, N. Garcia-Araez, C. P. Grey, *Angew. Chem. Int. Ed.* **2017**, *56*, 16057.
[2] Y. Zhang, W. Lv, Z. Huang, G. Zhou, Y. Deng, J. Zhang, C. Zhang, B. Hao, Q. Qi, Y.-B. He, F. Kang, Q.-H. Yang, *Sci. Bull.* **2019**, *64*, 910.
[3] C. Xia, C. Y. Kwok, L. F. Nazar, *Science* **2018**, *361*, 777.
[4] X. Gao, Y. Chen, L. Johnson, P. G. Bruce, *Nat. Mater.* **2016**, *15*, 882.
[5] S. E. Renfrew, B. D. McCloskey, *J. Am. Chem. Soc.* **2017**, *139*, 17853.
[6] D. Sharon, M. Afri, M. Noked, A. Garsuch, A. A. Frimer, D. Aurbach, *J. Phys. Chem. Lett.* **2013**, *4*, 3115.
[7] D. G. Kwabi, T. P. Batcho, C. V. Amanchukwu, N. Ortiz-Vitoriano, P. Hammond, C. V. Thompson, Y. Shao-Horn, *J. Phys. Chem. Lett.* **2014**, *5*, 2850.
[8] K. Takechi, T. Shiga, T. Asaoka, *Chem. Commun.* **2011**, *47*, 3463.
[9] S. R. Gowda, A. Brunet, G. M. Wallraff, B. D. McCloskey, *J. Phys. Chem. Lett.* **2013**, *4*, 276.
[10] H.-K. Lim, H.-D. Lim, K.-Y. Park, D.-H. Seo, H. Gwon, J. Hong, W. A. Goddard, III, H. Kim, K. Kang, *J. Am. Chem. Soc.* **2013**, *135*, 9733.
[11] W. Yin, A. Grimaud, F. Lepoivre, C. Yang, J. M. Tarascon, *J. Phys. Chem. Lett.* **2017**, *8*, 214.
[12] Z. Zhao, Y. Su, Z. Peng, *J. Phys. Chem. Lett.* **2019**, *10*, 322.
[13] S. Yang, P. He, H. Zhou, *Energy Environ. Sci.* **2016**, *9*, 1650.
[14] S. Zhang, M. J. Nava, G. K. Chow, N. Lopez, G. Wu, D. R. Britt, D. G. Nocera, C. C. Cummins, *Chem. Sci.* **2017**, *8*, 6117.
[15] T. Liu, J. P. Vivek, E. W. Zhao, J. Lei, N. Garcia-Araez, C. P. Grey, *Chem. Rev.* **2020**, *120*, 6558.
[16] L. Dong, L. Nie, W. Liu, *Adv. Mater.* **2020**, *32*, 1908494.
[17] B. Zhu, Y. Jin, X. Hu, Q. Zheng, S. Zhang, Q. Wang, J. Zhu, *Adv. Mater.* **2017**, *29*, 1603755.
[18] J. Wu, Z. Rao, X. Liu, Y. Shen, C. Fang, L. Yuan, Z. Li, W. Zhang, X. Xie, Y. Huang, *Adv. Mater.* **2021**, *33*, e2007428.
[19] K. Liao, S. Wu, X. Mu, Q. Lu, M. Han, P. He, Z. Shao, H. Zhou, *Adv. Mater.* **2018**, e1705711.
[20] Z. Huang, J. Ren, W. Zhang, M. Xie, Y. Li, D. Sun, Y. Shen, Y. Huang, *Adv. Mater.* **2018**, *30*, e1803270.
[21] Y. Xiao, R. Xu, C. Yan, Y. Liang, J.-F. Ding, J.-Q. Huang, *Sci. Bull.* **2020**, *65*, 909.
[22] Y. B. Zhang, W. Lv, Z. J. Huang, G. M. Zhou, Y. Q. Deng, J. Zhang, C. Zhang, B. Y. Hao, Q. Qi, Y. B. He, F. Y. Kang, Q. H. Yang, *Sci Bull* **2019**, *64*, 910.
[23] X. Shen, Y. Li, T. Qian, J. Liu, J. Zhou, C. Yan, J. B. Goodenough, *Nat. Commun* **2019**, *10*, 900.
[24] Y. Yu, Y.-B. Yin, J.-L. Ma, Z.-W. Chang, T. Sun, Y.-H. Zhu, X.-Y. Yang, T. Liu, X.-B. Zhang, *Energy Storage Mater.* **2019**, *18*, 382.
[25] W. Liang, F. Lian, N. Meng, J. Lu, L. Ma, C.-Z. Zhao, Q. Zhang, *Energy Storage Mater.* **2020**, *28*, 350.
[26] G. Wang, C. Chen, Y. Chen, X. Kang, C. Yang, F. Wang, Y. Liu, X. Xiong, *Angew. Chem. Int. Ed.* **2020**, *59*, 2055.
[27] T. Liu, X. L. Feng, X. Jin, M. Z. Shao, Y. T. Su, Y. Zhang, X. B. Zhang, *Angew Chem Int Edit* **2019**, *58*, 18240.
[28] M. Xie, X. Lin, Z. Huang, Y. Li, Y. Zhong, Z. Cheng, L. Yuan, Y. Shen, X. Lu, T. Zhai, Y. Huang, *Adv. Funct. Mater.* **2020**, *30*, 1905949.

[29] S. Wu, J. Yi, K. Zhu, S. Bai, Y. Liu, Y. Qiao, M. Ishida, H. Zhou, *Adv. Energy Mater.* **2017**, *7*, 1601759.
[30] Y. Ma, P. Qi, J. Ma, L. Wei, L. Zhao, J. Cheng, Y. Su, Y. Gu, Y. Lian, Y. Peng, Y. Shen, L. Chen, Z. Deng, Z. Liu, *Adv. Sci.* **2021**, *8*, e2100488.
[31] C. Wu, F. Guo, L. Zhuang, X. Ai, F. Zhong, H. Yang, J. Qian, *ACS Energy Lett.* **2020**, *5*, 1644.
[32] L. J. Li, X. S. Zhao, A. Manthiram, *Electrochem. Commun.* **2012**, *14*, 78.
[33] Y. Shen, D. Sun, L. Yu, W. Zhang, Y. Y. Shang, H. R. Tang, J. F. Wu, A. Y. Cao, Y. H. Huang, *Carbon* **2013**, *62*, 288.
[34] T. Zhang, H. S. Zhou, *Nat. Commun.* **2013**, *4*, 1817.
[35] X. F. Wang, D. Zhu, M. Song, S. R. Cai, L. Zhang, Y. G. Chen, *ACS Appl. Mater. Interfaces* **2014**, *6*, 11204.
[36] X. B. Zhu, T. S. Zhao, Z. H. Wei, P. Tan, L. An, *Energy Environ. Sci.* **2015**, *8*, 3745.
[37] Y. J. Liu, B. J. Li, H. Kitaura, X. P. Zhang, M. Han, P. He, H. S. Zhou, *ACS Appl. Mater. Interfaces* **2015**, *7*, 17307.
[38] F. C. Liu, Z. Shadike, X. F. Wang, S. Q. Shi, Y. N. Zhou, G. Y. Chen, X. Q. Yang, L. H. Weng, J. T. Zhao, Z. W. Fu, *Inorg. Chem.* **2016**, *55*, 6504.
[39] D. Safanama, S. Adams, *J. Power Sources* **2017**, *340*, 294.
[40] S. Wang, J. Wang, J. J. Liu, H. C. Song, Y. J. Liu, P. F. Wang, P. He, J. Xu, H. S. Zhou, *J. Mater. Chem. A* **2018**, *6*, 21248.
[41] Y. Liu, C. Li, B. Li, H. Song, Z. Cheng, M. Chen, P. He, H. Zhou, *Adv. Energy Mater.* **2018**, *8*, 1702374.
[42] Y. J. Liu, B. J. Li, Z. Cheng, C. Li, X. Y. Zhang, S. H. Guo, P. He, H. S. Zhou, *J. Power Sources* **2018**, *395*, 439.
[43] S. Song, X. Qin, Y. Ruan, W. Li, Y. Xu, D. Zhang, J. Thokchom, *J. Power Sources* **2020**, *461*, 228146.
[44] X. Chi, M. Li, J. Di, P. Bai, L. Song, X. Wang, F. Li, S. Liang, J. Xu, J. Yu, *Nature* **2021**, *592*, 551.
